# Dipropyleneglycol Dimethylether, New Green Solvent for Solid-Phase Peptide Synthesis: Further Challenges to Improve Sustainability in the Development of Therapeutic Peptides

**DOI:** 10.3390/pharmaceutics15061773

**Published:** 2023-06-20

**Authors:** Giovanni Vivenzio, Maria Carmina Scala, Pasquale Marino, Michele Manfra, Pietro Campiglia, Marina Sala

**Affiliations:** 1 Department of Pharmacy, University of Salerno, Via Giovanni Paolo II 132, Fisciano, 84084 Salerno, Italy; gvivenzio@unisa.it (G.V.); mscala@unisa.it (M.C.S.); 2 Department of Sciences, University of Basilicata, Via dell’Ateneo Lucano 10, 85100 Potenza, Italy; pasquale.marino@unibas.it (P.M.); michele.manfra@unibas.it (M.M.)

**Keywords:** green chemistry, solid-phase peptide synthesis, peptides, dipropyleneglycol dimethylether, recycling

## Abstract

In recent years, peptides have gained more success as therapeutic compounds. Nowadays, the preferred method to obtain peptides is solid-phase peptide synthesis (SPPS), which does not respect the principles of green chemistry due to the large number of toxic reagents and solvents used. The aim of this work was to research and study an environmentally sustainable solvent able to replace dimethylformamide (DMF) in fluorenyl methoxycarbonyl (Fmoc) solid-phase peptide synthesis. Herein, we report the use of dipropyleneglycol dimethylether (DMM), a well-known green solvent with low human toxicity following oral, inhalant, and dermal exposure and that is easily biodegradable. Some tests were needed to evaluate its applicability to all the steps of SPPS, such as amino acid solubility, resin swelling, deprotection kinetics, and coupling tests. Once the best green protocol was established, it was applied to the synthesis of different length peptides to study some of the fundamental parameters of green chemistry, such as PMI (process mass intensity) and the recycling of solvent. It was revealed that DMM is a valuable alternative to DMF in all steps of solid-phase peptide synthesis.

## 1. Introduction

Peptides play an important role in the fields of drug discovery and delivery, immunology, diagnostics, and biomaterials. Pharmaceutical industries are paying more and more attention to therapeutic peptides. In fact, there are currently more than 50 peptide drugs on the market and about 400 in the testing phase [1]. This relevance is attributed to their potential as drugs with high efficacy and minimal side effects [2]. SPPS is the preferred technique for the synthesis of therapeutic peptides, as it allows the synthesis of long-chain peptides, including those containing difficult sequence and cyclic peptides. Additionally, SPPS can be automated and is industrially scalable [3]. In recent years, the pressure to use less dangerous and more “benign” substances is growing, thanks to the work carried out by the ACS Green Chemistry Institute, which has drawn attention to greener processes development for the synthesis of biologically active peptides and to encourage much needed innovations [1]. In this context, the individual steps of the SPPS cannot be considered green due to the low atom economy (the presence of protecting groups) and mainly to a large amount of waste generated [2]. In fact, the main disadvantage of SPPS concerns the environmental pollution caused by a large number of toxic reagents and solvents used for all phases of peptide synthesis, particularly during the washing steps. The solvents most used in the SPPS are DMF, 1-Methyl-2-pyrrolidinone (NMP), and dichloromethane (DCM), which are substances that are not very safe for the environment, and humans and will soon need to be replaced [4]. Recently, researchers have identified more eco-sustainable solvents, such as 2-methyltetrahydrofuran (2-MeTHF) [5], tetrahydrofuran (THF) [6], cyclopentyl methyl ether (CPME) [7], γ-valerolactone (GVL) [8], N-formylmorpholine (NFM) [9], propylene carbonate (PC) [10], 1,3-Dimethyl-2-imidazolidinone (DMI) [11], N-butyl pyrrolidone (NBP) [12], 4-methyltetrahydropyran [13], dimethyl carbonate (DMC) [14], and cyclopentanone [15]. The most used coupling agents in SPPS are benzotriazole derivatives, which are considered dangerous and explosive. However, according to literature data, OxymaPure and COMU are safer reagents [4]. Unfortunately, green peptide chemistry is still a relatively small research field, and many of these advances take a long time to be applied to peptide manufacturing [1]. Taking this into consideration, in this work, we tested a new well-known green solvent, DMM [3], for all steps of the SPPS. DMM is a non-toxic solvent that is safe for the environment and humans and was first evaluated in terms of solubility, resin swelling, couplings, deprotections, and washing processes. After obtaining favorable results, the synthesis of a dipeptide was reported. Then, the best protocols were selected for the synthesis of two difficult sequences, such as Aib-enkephalin 5-mer and Aib-ACP 10-mer.

Various strategies have been suggested to tackle the cost challenges associated with eco-friendly SPPS solvents, including the increase in demand to lower costs and recycling. However, the high volume and low cost of conventional solvents pose obstacles to the widespread adoption of eco-friendly SPPS methods. As part of a green chemistry program focused on peptides, a more environmentally conscious approach to peptide circular chemistry, called “ReGreen SPPS”, has been proposed. This approach would use more-affordable and eco-friendly solvents and enable the easy recycling of reagents and solvents from SPPS waste [16].

## 2. Materials and Methods

### 2.1. Materials

Nα-Fmoc-protected amino acids, resins, coupling reagents, N,N-diisopropylethylamine (DIEA), piperidine, and trifluoroacetic acid (TFA) were purchased from Iris Biotech (Marktredwitz, Germany). Fmoc-Ala-Wang resin was purchased from Novabiochem^®^. Rink amide ChemMatrix resin was purchased from Biotage. Peptide-synthesis solvents and reagents, as well as acetonitrile (CH_3_CN) for HPLC, were reagent grade and were acquired from commercial sources and used without further purification unless otherwise noted.

#### Physical–Chemical Properties and Cost

The chemical–physical properties were taken from the technical data sheets provided by Sigma-Aldrich, and the price estimation was made based on the Merck price list that supplied us with the products (DMM and DMF).

### 2.2. Swelling Test

A total of 0.1 g for each of Trityl-OH ChemMatrix, Rink amide ChemMatrix, Wang ChemMatrix, Wang resin, Fmoc-Rink amide, 2-Chlorotritylchlor resin, TGS Wang, and TentaGel S-NH_2_ (Table S1) was weighed and placed in a reactor (10 mL) with a polypropylene filter occupying a volume of 0.4 mL. The desired solvent (DMF or DMM) was added to the resin and stirred at room temperature for 45 min. Subsequently, the solvent was removed from under vacuum, and the swelling degree was calculated according to this formula:

$$degree\;of\;swelling=\frac{volume\;of\;swollen\;resin-0.4\;mL}{0.1\;g}$$

Table S2 shows all the values of the three tests with their respective standard deviations and averages, while Figure S1 shows the volume of swollen resin.

### 2.3. Solubility Test

The solubility test was performed by adding 1 mL of DMM (or DMF) in 10 mL test tubes containing 0.1 mmol of Fmoc-AA-OH (or coupling agents), obtaining a concentration of 0.1 M. The tubes were placed on a shaker at room temperature until complete dissolution. In case of poor solubility, more solvent was added until complete dissolution, otherwise a greater quantity of AA was added until precipitation. The same was done with Fmoc-Val-OH and different coupling agents, starting from a concentration of 0.1 M. The figures of the test are reported in Figures S2 and S3, while Table S3 shows the solubilization efficacy of the Fmoc-AA(PG)-OH amino acids and coupling agents in DMF and DMM.

### 2.4. Deprotection Kinetics Test

Fmoc-Val-OH (0.1 mmol) was dissolved in a 20% piperidine/DMM solution. The reaction mixture was stirred at room temperature, and 10 µL were taken at time t = 0 (before base addition) and after 4, 6, 8, 10, 15, and 30 min. The samples were diluted with 1.5 mL of CH_3_CN/TFA (1% *v/v*) and injected into an HPLC-UV system. The same test was done with a 20% piperidine/DMF solution. The reaction was considered finished after the disappearance of the peak corresponding to Fmoc-Val-OH. Deprotection was monitored by analytical HPLC on an analytical column (Phenomex BioZen Peptide XB-C18 LC column, 150 × 2.1 mm, 2.6 µm) using a Shimadzu SPD 20 A UV/VIS detector, with detection at 220 nm. The column was perfused at a flow rate of 0.2 mL/min with solvent A (water in 0.1% aqueous TFA), and a linear gradient from 5 to 90% of solvent B (acetonitrile in 0.1% aqueous TFA) over 16 min was adopted for peptide elution. Chromatograms are reported in Figures S4–S16.

#### Evaluation of DBF and DBF-Base Adduct Formation

A total of 30 mg of Fmoc-Val-OH was dissolved in a 20% piperidine/DMM solution. The reaction mixture was performed under magnetic stirring at room temperature for 15 min. Then, the solvent was evaporated under vacuum, and the residue was dissolved in dichloromethane and concentrated under vacuum. The crude products were purified by preparative liquid chromatography (PLC) using the mixtures of n-hexane/ethyl acetate (9.5:0.5) as the mobile phase. TLC analysis was performed on precoated glass silica gel plates 60 (F254, 0.25 mm, VWR International).

The presence of DBF alone and the formation of the DBF-base adduct were directly analyzed by 1H NMR spectroscopy using a Bruker Avance (400 MHz) spectrometer (Figure S17).

### 2.5. Coupling Test: Synthesis of NH_2_-Tyr-Ala-OH

The dipeptide NH_2_-Tyr-Ala-OH was synthetized both manually and by microwave-assisted solid-phase peptide synthesis (MW-SPPS).

The manual synthesis was performed in polypropylene syringes fitted with a polyethylene porous disc, following a Fmoc/tBu strategy, on a Fmoc-Ala-Wang resin (50 mg, loading 0.71 mmol/g), previously swollen in DCM or DMM (1 × 45 min) at room temperature (rt). Deprotection of the resin Fmoc group was subsequently carried out with a 20% piperidine solution in DMF or DMM (1 × 5 min or 1 × 25 min, respectively). The resin was then washed after each step with DMF (2 × 1 min) and DCM (2 × 1 min) or DMM (2 × 1 min). Coupling with Fmoc-Tyr(tBu)-OH was performed manually using two pairs of coupling reagents: HOBt/DIC (1:1) or OxymaPure/DIC (1:2) for 2 h under stirring and at rt [17]. 

The Kaiser test was then applied to ensure proper coupling and deprotection [18]. Finally, after the dipeptide assembly, the N-terminal Fmoc group was removed and released from the resin using a cleavage mixture containing 95% of TFA, 2.5% triisopropylsilane (TIS), and 2.5% H_2_O for 2 h. The resin was removed by filtration, and the crude peptide was recovered by precipitation with cold anhydrous Et_2_O to give a white powder and then lyophilized.

For the microwave synthesis, a Biotage Initiator + Alstra automated microwave synthesizer was used [19]. Coupling reactions were achieved using Fmoc-Tyr(tBu)-OH (0.1 M), Oxyma (0.5 M), and DIC (0.5 M) in DMM at 75 °C (2×). After the dipeptide assembly, the cleavage was performed as above. The chromatograms and spectra are reported in Figures S18–S27.

### 2.6. Racemization Study

The Fmoc-Phenylglycine-Proline-NH_2_ (Fmoc-Phg-Pro-NH_2_) was synthesized using the new green protocol, as described above (OxymaPure/DIC 1:2, 3 eq. on Rink amide CM, 0.48 mmol/g), starting from Fmoc-L-Pro-OH and Fmoc-L-Phg-OH. In particular, the degree of racemization was studied on both the manually and the microwave-synthesized peptide. These data were compared to the data obtained when using DMF. The degree of racemization was calculated by analytical HPLC, and the chromatograms and spectra are reported in Figures S28–S32.

### 2.7. SPPS of Aib-Enkephalin and Aib-ACP

The peptides were synthesized as described above, using the new green protocol (OxymaPure/DIC 1:2, 3 eq., 2 × 10 min, 75° MW). Aib-enkephalin was synthesized using microwave-assisted synthesis on different resins (Wang PS and Rink amide CM), while Aib-ACP was synthesized on Rink amide CM resin. These data were compared to the data obtained when using DMF. The chromatograms and spectra are reported in Figures S33–S40.

#### Characterization

The degree of purity of NH_2_-Tyr-Ala-OH was determined using HPLC conditions in the following solvent system: A (water in 0.1% aqueous TFA) and CH_3_CN (acetonitrile in 0.1% aqueous TFA). The column (Phenomenex Aeris Peptide XB-C18, 150 × 4.6 mm, 3.6 µm) was perfused over 12 min at a flow rate of 1.5 mL/min using the following gradient: 0.01–10 min, 5–50% B, 10.01–11 min, 50–90%, 11.01–12 min, 90–5% B, using a Shimadzu SPD 20 A UV/VIS detector.

The degree of racemization of Fmoc-Phg-Pro-NH_2_ was determined using HPLC conditions in the above solvent system (solvents A and B) at a flow rate of 0.5 mL/min using the following gradient: 0.01–15 min, 5–50% B, 16.01–17 min, 50–95%, 17.01–21 min, 95–5% B, with C-18 column (BIOShell 160 A° Peptide C18, 100 × 2.1 mm, 2.6 µm).

The degree of purity of peptides was determined using HPLC conditions in the above solvent system (solvents A and B) at a flow rate of 1.5 mL/min using the following gradient: 0.01–10 min, 5–50% B, 10.01–11 min, 50–90%, 11–12 min, 90–5% B, with C-18 column (Phenomenex Aeris Peptide XB-C18, 150 × 4.6 mm, 3.6 µm).

The molecular weights of the peptides were determined by positive ESI infusion on LCMS IT-TOF (Shimadzu, Kyoto, Japan), while the molecular weight of NH_2_-Tyr-Ala-OH was determined by positive ESI infusion on an LTQ Orbitrap XL mass spectrometer (Thermo Scientific, Dreieich, Germany).

### 2.8. Solvent Recycling

The waste obtained from the synthesis of Aib-ACP decapeptide was collected and distilled at rt. It contained coupling, washing, and deprotection stream waste. A simple distillation apparatus was used for the distillation with a Liebig condenser (300 mm). The green solvent, DMM, was recovered with a yield of 80% of all solvent used [20]. The green solvent recycled was analyzed by ^1^H NMR spectroscopy and compared to the new one (Figure S41 and Tables S4–S7).

## 3. Results and Discussion

Solid-phase peptide synthesis has a low score on the following green chemistry metrics: atom economy, environmental (E) factor, and process mass intensity (PMI) [21], so the goal of this work is the search for a solvent that might reflect the parameters of green chemistry and, at the same time, apply to all phases of peptide synthesis. As reported by Al Musaimi et al. [3], DMM is a green solvent with low human toxicity following oral, inhalant, and dermal exposure and easily biodegradable. This solvent, in fact, according to Regulation (EC) N. 1272/2008, is classified as a non-hazardous substance.

First, the chemical–physical properties of DMM are compared to the solvent most used in SPPS, DMF, such as viscosity and boiling point (Table 1). Viscous substances are too much trouble during the filter stage since they may not get through a polypropylene filter. Additionally, low-boiling substances are trouble because they are dangerous during microwave synthesis. DMM has a viscosity slightly higher than DMF, but this does not compromise passage through the filter. The boiling point of DMM is higher than that of DMF, making the selected green solvent also applicable to microwave-assisted synthesis. As shown in Table 1, the green solvent is much more expensive than DMF, but cost reduction could be achieved through global supply by companies and academies and through recycling.

### 3.1. Resin Swelling Studies: Swelling Test

**Figure 1:**
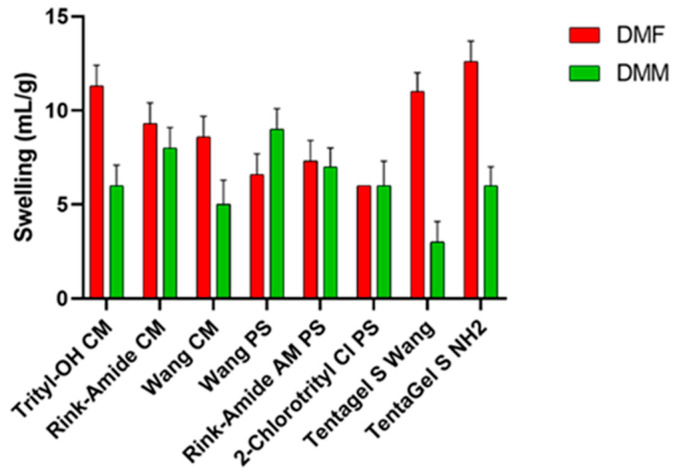
Swelling results of several resins in DMF and DMM.

A good solvent should adequately swell a variety commercially available resins, demonstrating broad applicability to SPPS. The choice of resin is crucial because, along with the linker, it determines the functional group of the C-terminal and the synthetic strategy [22,23]. For this test, different resins were selected: resins based on polystyrene (PS), polyethylene glycol (PEG) resins functionalized with PS, and resins consisting 100% of PEG. Polystyrene resins contain 1–2% divinyl benzene (DVB), which forms a lattice between polystyrene monomers and provides rigidity to the resin itself and better swell in non-polar solvents. TentaGel (TG) resins consist of PEG (50–70%) bonded to a low cross-linking polystyrene base and swell in both polar and non-polar solvents. Finally, ChemMatrix, a totally PEG resin, has better swelling properties in most of the solvents used in SPPS [24], making it suitable for microwave-assisted synthesis. During the swelling test (Figure 1), resin was introduced in a syringe and swelled for 45 min under shaking with the selected solvent (DMF or DMM). Then, the solvent was removed from under vacuum, and the swelling degree was evaluated using the formula described above. These values were compared to the values obtained when using DMF. As shown in Figure 1, TentaGel resins, Trityl, and Wang ChemMatrix show a lower degree of swelling in the green solvent, while Rink amide resins and the 2-chlorotrityl resin have almost the same degree of swelling in both solvents. Finally, DMM is able to swell Wang PS resin better than DMF. Ethers are unresponsive substances, making them good solvents for reactions. Most ethers are polar, but DMM has a relatively high molecular weight, giving it both hydrophilic and lipophilic properties. This may explain why it is able to swell polystyrene-based resins.

The test was performed in triplicate, and results show the average of the three tests. The standard deviation for each measurement is reported in Table S3.

### 3.2. Solubility Test

To assess the efficiency of the selected solvents during SPPS, we evaluated their capability to solubilize amino acids and coupling agents. Furthermore, to avoid the formation of side products, we used amino acids protected both in the side chain and on the α-amino function. The protecting groups, therefore, constitute an important obstacle to solubility. During this test, we solubilized amino acids protected on the amino function with the base-labile Fmoc groups and in the side chain with the acid-labile groups. Starting from the standard amino acid concentration for SPPS of 0.1 M in DMM, we carried out dilutions in case of incomplete dissolution or concentrations up to the formation of precipitate. Table 2 lists the solubilities of the amino acids in DMM and DMF; only Fmoc-His(Trt)-OH showed solubility in DMM at a lower concentration than the standard concentration for SPPS.

A solvent mixture was used to address the issue of poor solubility of histidine. Mixing solvents can improve certain chemical and physical properties, such as viscosity and density, thereby enhancing the efficacy of solubilization. Unfortunately, it is not always possible to create completely green mixtures. In fact, in most cases, one of the two solvents does not meet any green chemistry parameters. To improve the solubility of histidine in DMM, we tested different concentrations, ranging from 0.01 M to 0.1 M, with varying percentages of NMP (Table 3). At the standard SPPS concentration of 0.1 M, histidine is soluble with a 40% NMP/DMM mixture.

Table 4 also shows the solubility values of the coupling agents in DMM and DMF. Most of the coupling reagents exhibit good solubility in DMM comparable to the standard concentration (0.1 M), except COMU and HATU, which are insoluble. Then, Fmoc-Val-OH and Fmoc-His(Trt)-OH were mixed with coupling agents (HOBt/DIC and Oxyma/DIC), and the solubility was evaluated (Table 4). As shown in Table 4, Fmoc-His(Trt)-OH showed better solubility in combination with coupling agents, reducing the percentage of NMP (from 40% to 30%). In regard to the coupling agents, different chemical classes lead to the formation of the amide bond. The most used are derivatives of benzotriazole (hydroxybenzotrazole or HOBt and 2-1H-Benzotriazole-1-yl-1,1,3,3-tetramethyluronium hexafluorophosphate or HBTU) and are usually coupled to carbodiimides. Benzotriazole derivatives are unsafe for humans and the environment. The derivatives of ethyl cyanoacetate, such as the oxime of ethyl cyanoacetate (OxymaPure), are activators that neutralize the basicity of carbodiimides, thanks to their acidic character (pka 4.6), thus preventing the catalyzed base reactions, in particular the racemization. Furthermore, OxymaPure, used as additive in the carbodiimide (DIC) approach, allows the desired product with quantitative yields and is fully compatible with microwave-assisted peptide synthesis [25].

### 3.3. Deprotection Kinetics Test

**Figure 2:**
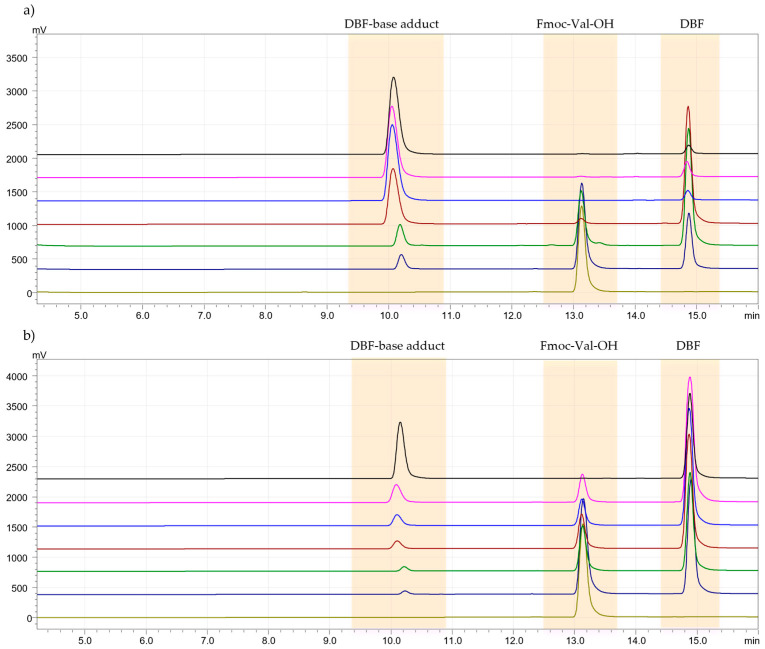
(**a**). Chromatogram of Fmoc-Val-OH deprotection kinetics test with the 20% solution of piperidine in DMF. Legend: t_0_ = 0 min (light green), t_1_ = 2 min (blue), t_2_ = 4 min (green), t_3_ = 6 min (red), t_4_ = 8 min (light blue), t_5_ = 15 min (pink), t_6_ = 30 min (black). (**b**). Chromatogram of Fmoc-Val-OH deprotection kinetics test with the 20% solution of piperidine in DMM. Legend: t_0_ = 0 min (light green), t_1_ = 2 min (blue), t_2_ = 4 min (green), t_3_ = 6 min (red), t_4_ = 8 min (light blue), t_5_ = 15 min (pink), t_6_ = 30 min (black).

Generally, in solid-phase peptide synthesis (SPPS), a solution of piperidine in DMF is used for removing the Fmoc group from the α-amino function. In this study, a deprotection kinetics test was conducted to evaluate the time required by the solvent, DMM, in combination with piperidine, to remove the Fmoc group and to assess any side reactions that may have occurred between the solvent and the base used [26]. The test was performed with a 20% solution of piperidine in DMM, and the formation of dibenzofulvene (DBF) was monitored by HPLC-UV at times 0, 4, 6, 8, 15, and 30 min. The deprotection kinetics were compared to the method developed by Jad et al. [27]. For both protocols, Fmoc-Val-OH was examined. With the standard solution of 20% piperidine in DMF, the peak relative to Fmoc-Val-OH disappeared after 6 min, while with the green solvent (piperidine at 20% in DMM), after 30 min (Figure 2a,b). Although the times are longer, they are acceptable for a deprotection reaction. Certainly, DMM does not bring side reactions, and this step of SPPS has been improved in terms of eco-sustainability. DBF and DBF-base adduct were characterized by 1H-NMR spectroscopy in Figure S17.

### 3.4. Coupling Test

We evaluated the effectiveness of the green solvent during the synthesis of dipeptide NH_2_-Tyr-Ala-OH. The synthesis of this peptide was performed in DMF and DMM, screening two pairs of coupling agents. After swelling Fmoc-Ala-Wang resin, for 45 min, all the steps of the SPPS were performed: Fmoc deprotection, coupling, and washing. For this test, two pairs of coupling agents were selected: non-green (HOBt/DIC, entries 1 and 2) and greener coupling agents (OxymaPure/DIC, entries 3 and 4) [28,29]. After cleavage, the crude dipeptide was analyzed by HPLC, and the degree of purity was quantified by integration of the peaks. Results are shown in Table 5. Entries 1 and 2 show that, under these conditions, the highest values of purity were obtained with DMM. Moreover, the results indicated that, when using greener coupling agents (entries 3 and 4), the percentage of purity was lower when DMF was used during the coupling step. Considering these results, the combination of OxymaPure/DIC was considered the best method for further investigations. Therefore, in order to further optimize the protocol and make it scalable to industry, it was applied to microwaves (entry 5). Entry 5 shows a result quite similar in comparison to the synthesis performed at room temperature. This method was used to synthesize the longer peptides.

### 3.5. Racemization Study

Racemization is one of the primary collateral reactions of peptides. This reaction can be catalyzed by strong bases, such as NaOH, but can also occur with weak organic bases, such as piperidine or DIPEA (N, N-Diisopropylethylamine) or basic coupling agents, such as DIC. To evaluate the efficiency of new protocols, the degree of racemization was assessed during the synthesis of dipeptide Fmoc-Phg-Pro-NH_2_. This peptide is particularly prone to racemization due to the acidity of the benzylic alpha-proton in the side chain of Phg [30]. It is known that high temperatures can promote racemization. Therefore, the synthesis was carried out both in the selected green solvent and in DMF, and the same manual synthesis protocol was compared to the microwave-assisted one. Table 6 shows the degree of racemization with the different protocols. Despite microwaves being able to promote racemization, the DL degree of the dipeptide synthesized in DMM is significantly lower than that in DMF (entry 9). This good result could allow the synthesis of sequences containing amino acids that are prone to racemization, at medium-high temperatures.

### 3.6. SPPS of Aib-Enkephalin and Aib-ACP

After testing the green solvent, DMM, in all stages of SPPS, we evaluated the effectiveness of the solvent during the synthesis of a pentapeptide, Aib-enkephalin (H-Tyr-Aib-Aib-Phe-Leu-OH/NH_2_). This sequence represents a difficult step due to the presence of two Aib (2-Aminoisobutyric acid) residues, which are sterically hindered amino acids and often lead to incomplete or slow-rate coupling reactions. In fact, the misincorporation of Aib residues (des-Aib) could easily happen during the synthesis [5]. The amount of des-Aib measures the efficiency of DMM compared to DMF. Aib-enkephalin was synthesized using the microwave-assisted synthesis technique on different resins, particularly those that had shown good swelling: Wang PS and Rink amide CM. The new green protocol previously selected was applied to the synthesis of the pentapeptide, and DMM was used in all stages, including washing. Purities of pentapeptide were calculated by HPLC and are shown in Table 7.

As the Aib-aib coupling is considered the key step of the synthesis, the percentage of des-Aib was calculated. When the synthesis was performed on a Wang resin, using DMM (entry 11), a lower percentage of pentapeptide was obtained compared to DMF, but it gave a much higher des-aib rate than the one in DMF. Interestingly, results obtained on a Rink amide CM resin (entry 13), using DMM, are better than those obtained in DMF (entry 12). For both syntheses, no amount of des-Aib was observed. As consequence, the Rink amide CM was selected for further studies. The same protocol was applied to the synthesis of a longer and more difficult sequence, Aib-ACP decapeptide (H-Val-Gln-Aib-Aib-Ile-Asp-Tyr-Ile-Asn-Gly-NH_2_), used as a case study due to its tendency for aggregation and folding during SPPS [16]. HPLC purities of Aib-ACP are shown in Table 8.

In this case, the synthesis of the decapeptide in DMM (entry **15**) yielded purity values compared to DMF (entry **14**), with a small amount of des-Aib observed for entry **15**. These excellent results, obtained for both the synthesis of Aib-enkephalin and the decapeptide Aib-ACP, make DMM an ideal greener alternative to DMF.

### 3.7. Solvent Recycling

Although DMM has proven to be a valid alternative to DMF, SPPS cannot be considered green due to the high costs of the solvent. One solution to reduce waste, costs, and PMI (process mass intensity) is solvent recovery. PMI is a parameter that measures the sustainability of a process. It considers all the materials used in a process and can be easily calculated using the ratio between the total mass of materials and the mass of the isolated product [31,32]. DMM can be easily recovered by distillation, considering that its boiling point is quite different from that of the other volatile components (DMM: 175 °C, DIC: 145 °C, and piperidine: 106 °C). The green solvent recycled was directly analysed by 1H NMR spectroscopy (Figure S41) [32]. The recovered DMM can be used for further synthesis. Table 9 shows the PMI values of DMM before and after solvent recovery. Through recycling, it decreased from 3750.20 to 1370.68. These important data can certainly balance the high cost of DMM compared to those of DMF.

## 4. Conclusions

In this work, we studied DMM as a green alternative to traditional solvents used in SPPS. This ether has shown good ability to solubilize amino acids and coupling agents, as well as good capability to swell some commercially available resins and to remove the Fluorenylmethyloxycarbonyl protecting group. It was tested first during a small peptide synthesis and then on a larger one, obtaining excellent results. DMM also showed full applicability in microwave-assisted peptide synthesis, demonstrating that the green solvent could be a valid alternative to hazardous DMF in all processes of SPPS. Based on these findings, we intend to apply the developed protocol to our studies in order to evaluate its potential for the large-scale production of therapeutic peptides. Certainly, the cost of this solvent is high, so recovery and recycling are considered and studied to reduce the PMI and make SPPS green.

## Figures and Tables

**Table 1:** Physical–chemical properties and cost of DMF and DMM.

Physical–Chemical Properties	Solvent	
	**DMF**	**DMM**
Boiling point	153 °C	175 °C
Density	0.95 g/cm^3^	0.90 g/cm^3^
Viscosity	0.85 mm^2^/s	1.12 mm^2^/s
Log P	−0.85	0.42
Melting point	−31 °C	−80 °C
Flash point	58 °C	65 °C
Cost	72.50 Eur/liter	128.00 Eur/liter

**Table 2:** Solubilization efficacy of Fmoc-AA(PG)-OH amino acids in DMF and DMM ^a^.

Solvent	Fmoc-aa(PG)-OH																			
	Gly	Ile	Leu	Val	Ala	Phe	Pro	Met	Tyr (tBu)	Thr (tBu)	Ser (tBu)	Asp (OtBu)	Glu (OtBu)	Cys (Trt)	Asn (Trt)	Gln (Trt)	His (Trt)	Lys (Boc)	Trp (Boc)	Arg (Pbf)
**DMF**																				
**DMM**																				

^a^ Solubilization monitored at 0.1 M concentration. Legend: green = soluble; red = insoluble. PG = protecting group.

**Table 3:** Solubility of Fmoc-His(Trt)-OH amino acid in mixture NMP/DMM ^a^.

Concentration (M)	NMP (%)			
0.1	10	20	30	40
			

^a^ Legend: green = soluble, red = insoluble.

**Table 4:** Solubilization efficacy of coupling reagents and mixture Fmoc-AA(PG)-OH with coupling reagents (A–B) in DMF and DMM ^a^.

	Coupling Reagents								Mixture Fmoc-Val-OH + Coupling Reagents (A–B)		Mixture Fmoc-His(Trt)-OH + Coupling Reagents (A–B)	
Solvent	HOBt	CDI	Oxyma Pure	HATU	COMU	DIC	HOBt/DIC (A)	OxymaPure/DIC (B)	Val + A	Val + B	His (Trt) + A	His (Trt) + B
DMF												
DMM											30% NMP	30% NMP

^a^ Legend: green = soluble, red = insoluble.

**Table 5:** Synthesis of NH_2_-Tyr-Ala-OH dipeptide with different approaches.

Entry	Solvent	Coupling Agents	Eq.	Time	T (°C)	Purity (%)
1	DMF	HOBt/DIC (1:1)	3	2h	rt	79.68
2	DMM	HOBt/DIC (1:1)	3	2h	rt	91.14
3	DMF	OxymaPure/DIC (1:2)	3	2h	rt	33.65
4	DMM	OxymaPure/DIC (1:2)	3	2h	rt	83.57
5	DMM	OxymaPure/DIC (1:2)	3	10 min	75 (MW)	78.90

**Table 6:** Racemization ratio (%) in the synthesis of Fmoc-Phg-Pro-NH_2_ ^a^.

Entry	Solvent	Time	T (°C)	DL ^b^ (%)
6	DMF	2 h	rt	48.78
7	DMM	2 h	rt	65.80
8	DMF	10 min	75 (MW)	89.02
9	DMM	10 min	75 (MW)	35.29

^a^ Racemization was calculated by HPLC. ^b^ Defined as (Fmoc-D-Phg-Pro-NH_2_/Fmoc-L-Phg-Pro-NH_2_) × 100.

**Table 7:** HPLC purities of Aib-enkephalin pentapeptide assembled on different resins.

Entry	Solvent	Resin	Pentapeptide (%)	Des-Aib (%)	Other (%)
10	DMF	Wang PS	89.20	1.39	9.41
11	DMM	Wang PS	81.76	8.65	9.59
12	DMF	Rink amide CM	94.88	-	5.12
13	DMM	Rink amide CM	95.60	-	4.40

**Table 8:** HPLC purities of Aib-ACP decapeptide assembled on Rink amide CM.

Entry	Solvent	Decapeptide (%)	Des-Aib (%)	Other (%)
14	DMF	53.73	-	46.27
15	DMM	53.69	9.24	37.07

**Table 9:** Process mass intensity (PMI) of synthesis and recovery ^a^.

Entry	Solvent	PMI	Recovery (Yield %)	PMI after Recovery
1	DMF	3896.28	-	-
2	DMM	3750.20	80	1370.68

^a^ The wastes coming from the coupling, washing, and deprotection steps were distilled together.

## Data Availability

The data presented in this study are available on request from the corresponding author.

## Supplementary Materials

The following supporting information can be downloaded at https://www.mdpi.com/article/10.3390/pharmaceutics15061773/s1, Table S1: Solubilization efficacy of Fmoc-AA(PG)-OH amino acids and coupling agents in DMF and DMM; Figures S1 and S2: Solubilization efficacy of Fmoc-AA(PG)-OH amino acids and coupling agents in DMM; Table S2: Physical parameters of resins evaluated for swelling tests; Figure S3: Swelling of several resins in DMM and DMF; Table S3: Calculations of standard deviations for the values of the swelling tests in triplicate; Figure S4–S11: Chromatogram of Fmoc-Val-OH deprotection in DMM at t = 0, 2, 4, 6, 8, 10, 15 and 30 min; Figure S12–S16: Chromatogram of Fmoc-Val-OH deprotection in DMF at t = 0, 2, 4, 6 and 8 min; Figure S17: 1H NMR spectrum (400 MHz, DCM-d6) after deprotection with 20% piperidine in DMM of Fmoc-Val-OH; Figure S18–S26: Chromatogram of NH_2_-Tyr-Ala-OH dipeptide; Figure S27: ESI-MS of NH_2_-Tyr-Ala-OH dipeptide ion [M + H]+ (253.0833 Da); Figure S28–S31: Chromatogram of Fmoc-Phg-Pro-NH_2_ dipeptide; Figure S32: ESI-MS of Fmoc-Phg-Pro-NH_2_ dipeptide ion [M + H]+ (470.2083 Da); Figure S33–S36: Chromatogram of Aib-enkephalin; Figure S37: ESI-MS of Aib- enkephalin ion [M + H]+ (611.3532 Da); Figures S38 and S39: Chromatogram of Aib-ACP; Figure S40: ESI-MS of Aib-ACP ion [M + H]+ (1090.5859 Da); Tables S4–S7: Overview of starting materials used for decapeptide (Aib-ACP), their total mass and PMI before and after recycling; Figure S41: (a) ^1^H NMR spectrum (CD_2_Cl_2_, 400 MHz, 298 K) of DMM recovered from distillation processes; (b) ^1^H NMR spectrum of DMM.

## References

[1] Isidro-Llobet A., Kenworthy M.N., Mukherjee S., Kopach M.E., Wegner K., Gallou F., Smith A.G., Roschangar F. (2019). Sustainability Challenges in Peptide Synthesis and Purification: From R&D to Production. J. Org. Chem..

[2] Martin V., Egelund P.H.G., Johansson H., Le Quement S.T., Wojcik F., Sejer Pedersen D. (2020). Greening the synthesis of peptide therapeutics: An industrial perspective. RSC Adv..

[3] Al Musaimi O., de la Torre B.G., Albericio F. (2020). Greening Fmoc/tBu solid-phase peptide synthesis. Green Chem..

[4] Jad Y.E., Kumar A., El-Faham A., de la Torre B.G., Albericio F. (2019). Green Transformation of Solid-Phase Peptide Synthesis. ACS Sustain. Chem. Eng..

[5] Jad Y.E., Acosta G.A., Govender T., Kruger H.G., El Faham A., de la Torre B.G., Albericio F. (2016). Green Solid-Phase Peptide Synthesis 2. 2-Methyltetrahydrofuran and Ethyl Acetate for Solid-Phase Peptide Synthesis under Green Conditions. ACS Sustain. Chem. Eng..

[6] Jad Y.E., Acosta G.A., Khattab S.N., de la Torre B.G., Govender T., Kruger H.G., El-Faham A., Albericio F. (2015). Peptide synthesis beyond DMF: THF and ACN as excellent and friendlier alternatives. Org. Biomol. Chem..

[7] Jad Y.E., Acosta G.A., Khattab S.N., de la Torre B.G., Govender T., Kruger H.G., El-Faham A., Albericio F. (2016). 2-Methyltetrahydrofuran and cyclopentyl methyl ether for green solid-phase peptide synthesis. Amino Acids.

[8] Kumar A., Jad Y.E., Collins J.M., Albericio F., de la Torre B.G. (2018). Microwave-Assisted Green Solid-Phase Peptide Synthesis Using γ-Valerolactone (GVL) as Solvent. ACS Sustain. Chem. Eng..

[9] Kumar A., Jad Y.E., El-Faham A., de la Torre B.G., Albericio F. (2017). Green solid-phase peptide synthesis 4. γ-Valerolactone and N-formylmorpholine as green solvents for solid phase peptide synthesis. Tetrahedron Lett..

[10] Lawrenson S.B., Arav R., North M. (2017). The greening of peptide synthesis. Green Chem..

[11] Byrne F.P., Jin S., Paggiola G., Petchey T.H.M., Clark J.H., Farmer T.J., Hunt A.J., McElroy C.R., Sherwood J. (2016). Tools and techniques for solvent selection: Green solvent selection guides. Sustain. Chem. Process..

[12] Lopez J., Pletscher S., Aemissegger A., Bucher C., Gallou F. (2018). N-Butylpyrrolidinone as Alternative Solvent for Solid-Phase Peptide Synthesis. Org. Process Res. Dev..

[13] Nienałtowski T., Krzesiński P., Baumert M.E., Skoczeń A., Suska-Kauf E., Pawłowska J., Kajetanowicz A., Grela K. (2020). 4-Methyltetrahydropyran as a Convenient Alternative Solvent for Olefin Metathesis Reaction: Model Studies and Medicinal Chemistry Applications. ACS Sustain. Chem. Eng..

[14] Schäffner B., Schäffner F., Verevkin S.P., Börner A. (2010). Organic carbonates as solvents in synthesis and catalysis. Chem. Rev..

[15] Liu Y., Chen Z., Wang X., Liang Y., Yang X., Wang Z. (2017). Facile Synthesis of N,S-Codoped Hierarchically Porous Carbon with High Volumetric Pseudocapacitance. ACS Sustain. Chem. Eng..

[16] Pawlas J., Rasmussen J.H. (2019). ReGreen SPPS: Enabling circular chemistry in environmentally sensible solid-phase peptide synthesis. Green Chem..

[17] Orlandin A., Guryanov I., Ferrazzano L., Biondi B., Biscaglia F., Storti C., Rancan M., Formaggio F., Ricci A., Cabri W. (2022). Carbodiimide-Mediated Beckmann Rearrangement of Oxyma-B as a Side Reaction in Peptide Synthesis. Molecules.

[18] Kaiser E., Colescott R., Bossinger C., Cook P. (1970). Color test for detection of free terminal amino groups in the solid-phase synthe-sis of peptides. Anal. Biochem..

[19] Sala M., Spensiero A., Esposito F., Scala M.C., Vernieri E., Bertamino A., Manfra M., Carotenuto A., Grieco P., Novellino E. (2016). Development and Identification of a Novel Anti-HIV-1 Peptide Derived by Modification of the N-Terminal Domain of HIV-1 Integrase. Front. Microbiol..

[20] Martelli G., Cantelmi P., Tolomelli A., Corbisiero D., Mattellone A., Ricci A., Fantoni T., Cabri W., Vacondio F., Ferlenghi F. (2021). Steps towards sustainable solid phase peptide synthesis: Use and recovery of N-octyl pyrrolidone. Green Chem..

[21] Ballester-Caudet A., Campins-Falcò P., Pèrez B., Sancho R., Lorente M., Sastre G., Gonzàlez C. (2019). A new tool for evaluating and/or selecting analytical methods: Summarizing the information in a hexagon. Trends Anal. Chem..

[22] Hudson D. (1999). Matrix assisted synthetic transformations: A mosaic of diverse contributions. I. The pattern emerges. J. Comb. Chem..

[23] Hudson D. (1999). Matrix assisted synthetic transformations: A mosaic of diverse contributions. II. The pattern is completed. J. Comb. Chem..

[24] Garcia-Martin F., Quintanar-Audelo M., Garcia-Ramos Y., Cruz L.J., Gravel C., Furic R., Cruz S., Tulla-Puche J., Albericio F. (2006). ChemMatrix, a Poly(ethylene glycol)-Based Support for the Solid-Phase Synthesis of Complex Peptides. J. Comb. Chem..

[25] El-Faham A., Subirós Funosas R., Prohens R., Albericio F. (2009). COMU: A safer and more effective replacement for benzotriazole-based uronium coupling reagents. Chemistry.

[26] Luna O.F., Gomez J., Cárdenas C., Albericio F., Marshall S.H., Guzmán F. (2016). Deprotection Reagents in Fmoc Solid Phase Peptide Synthesis: Moving Away from Piperidine?. Molecules.

[27] Carpino L.A., Han G.Y. (1972). The 9-fluorenylmethoxycarbonyl amino-protecting group. J. Org. Chem..

[28] Valeur E., Bradley M. (2009). Amide bond formation: Beyond the myth of coupling reagents. Chem. Soc. Rev..

[29] Subirós-Funosas R., Prohens R., Barbas R., El-Faham A., Albericio F. (2009). Oxyma: An Efficient Additive for Peptide Synthesis to Replace the Benzotriazole-Based HOBt and HOAt with a Lower Risk of Explosion. Chem. Eur. J..

[30] Liang C., Behnam M.A., Sundermann T.R., Klein C.D. (2017). Phenylglycine racemization in Fmoc-based solid-phase peptide synthesis: Stereochemical stability is achieved by choice of reaction conditions. Tetrahedron Lett..

[31] Fantoni T., Tolomelli A., Cabri W. (2022). A translation of the twelve principles of green chemistry to guide the development of cross-coupling reactions. Catal. Today.

[32] Mattellone A., Corbisiero D., Ferrazzano L., Cantelmi P., Martelli G., Palladino C., Tolomelli A., Cabri W. (2023). Speeding up sustainable solution-phase peptide synthesis using T3P® as a green coupling reagent: Methods and challenges. Green Chem..

