# Supplementary material for: Dipropyleneglycol Dimethylether, New Green Solvent for Solid-Phase Peptide Synthesis: Further Challenges to Improve Sustainability in the Development of Therapeutic Peptides
Source: Pharmaceutics. 2023 Jun 20;15(6):1773. doi: 10.3390/pharmaceutics15061773 (PMC10304503; doi:10.3390/pharmaceutics15061773)

SUPPLEMENTARY MATERIAL

Dipropyleneglycol Dimethylether, New Green Solvent for Solid-Phase Peptide Synthesis: Further Challenges to Improve Sustainability in the Development of Therapeutic Peptides

Giovanni Vivenzio [1,†], Maria Carmina Scala [1,†], Pasquale Marino [2], Michele Manfra [2], Pietro Campiglia [2,*] and Marina Sala [1,*]

[1] Department of Pharmacy, University of Salerno, Via Giovanni Paolo II 132, 84084 Fisciano, Salerno, Italy

[2] Department of Sciences, University of Basilicata, Via dell'Ateneo Lucano 10, 85100 Potenza, Italy

*** Corresponding authors:**

Pietro Campiglia,

Phone: +39-089969242, E-mail: pcampiglia@unisa.it;

Marina Sala,

Phone: +39-089968148, E-mail: msala@unisa.it.

† These authors contributed equally to this work.

Table of contents:

Swelling tests.. S2
Solubility tests.. S3
Deprotection kinetics test S5
Coupling test S12
Racemization study S17
Green SPPS S19
Recycling of decapeptide SPPS waste stream S22

Swelling tests

Table S1. Physical parameters of resins evaluated for swelling tests.

Resin	Loading (mmol·g-1)	Bead Size (Mesh)	Cross-Linking (DVB %)
Wang-ChemMatrix	0.60	35-100	-
Trityl-OH-ChemMatrix	0.44	35-100	-
Rink-Amide-ChemMatrix	0.48	35-100	-
Rink Amide AM PS	0.64	100-200	1
Wang resin PS	0.30	100-200	1
2-Chlorotrityl chloride resin PS	1.55	200-400	1
TentaGel S NH2	0.26	75-150	-
TentaGel S Wang	0.26	170	-

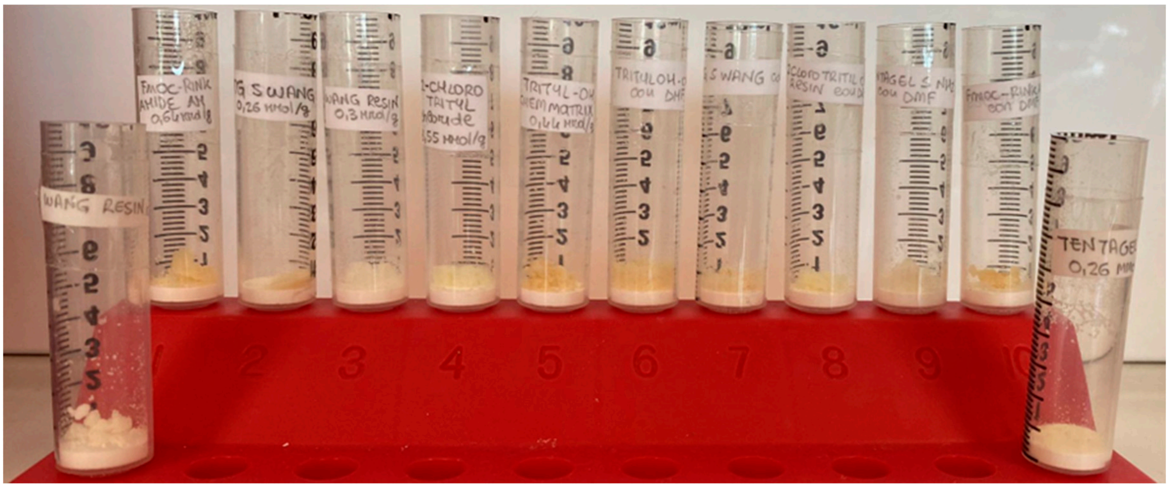

Figure S1. Swelling of several resins in DMM and DMF.

Table S2. Calculations of standard deviations for the values of the swelling tests in triplicate.

	DMM					DMF				
	Swell1	Swell2	Swell3	Mean	SD	Swell1	Swell2	Swell3	Mean	SD
2-Chlorotrityl chloride PS	8	6	5.5	6.5	1.3	6	6	6	6	0
Fmoc-Rink Amide AM PS	8	6	7	7	1.0	8	6	8	7.333	1.1
Wang resinPS	10	8	10	9.333	1.1	8	6	6	6.667	1.1
TentaGel S Wang	2	4	4	3.333	1.1	12	10	11	11	1.0
TentaGel S NH2	6.5	6	8	6.833	1.0	14	12	12	12.667	1.1
Wang ChemMatrix	6.5	4	6	5.5	1.3	8	8	10	8.667	1.1

Rink-Amide ChemMatrix	10	8	8	8.667	1.1	10	8	10	9.333	1.1
Trityl-OH ChemMatrix	6	8	10	8	1.1	12	10	12	11.333	1.1

Solubility tests

Table S3. Solubilization efficacy of Fmoc-AA(PG)-OH amino acids and coupling agents in DMF and DMM. Solubilization monitored at 0.1 M concentration.

Reagent	Solubility (M)	
	**DMF**	**DMM**
Fmoc-Gly-OH	>0.9	0.17
Fmoc-Ile-OH	>0.9	0.41
Fmoc-Leu-OH	>0.9	0.5
Fmoc-Val-OH	>0.9	0.45
Fmoc-Ala-OH	>0.9	0.22
Fmoc-Phe-OH	0.35	0.15
Fmoc-Pro-OH	>0.9	0.45
Fmoc-Met-OH	>0.9	0.31
Fmoc-Tyr(tBu)-OH	0.9	0.2
Fmoc-Thr(tBu)-OH	>0.9	>0.9
Fmoc-Ser(tBu)-OH	>0.9	>0.9
Fmoc-Asp(OtBu)-OH	>0.9	0.15
Fmoc-Glu(OtBu)-OH	>0.9	>0.9
Fmoc-Cys(Trt)-OH	0.45	0.13
Fmoc-Asn(Trt)-OH	>0.9	0.1
Fmoc-Gln(trt)-OH	>0.9	0.18
Fmoc-His(Trt)-OH	0.3	0.01
Fmoc-Lys(Boc)-OH	>0.9	0.26
Fmoc-Trp(Boc)-OH	0.9	>0.9
Fmoc-Arg(Pbf)-OH	>0.9	>0.9
HOBt	>0.9	0.16
CDI	>0.9	0.4
OxymaPure	>0.9	>0.9
HATU	0.5	Ins.
COMU	>0.9	Ins.
DIC	>0.9	>0.9

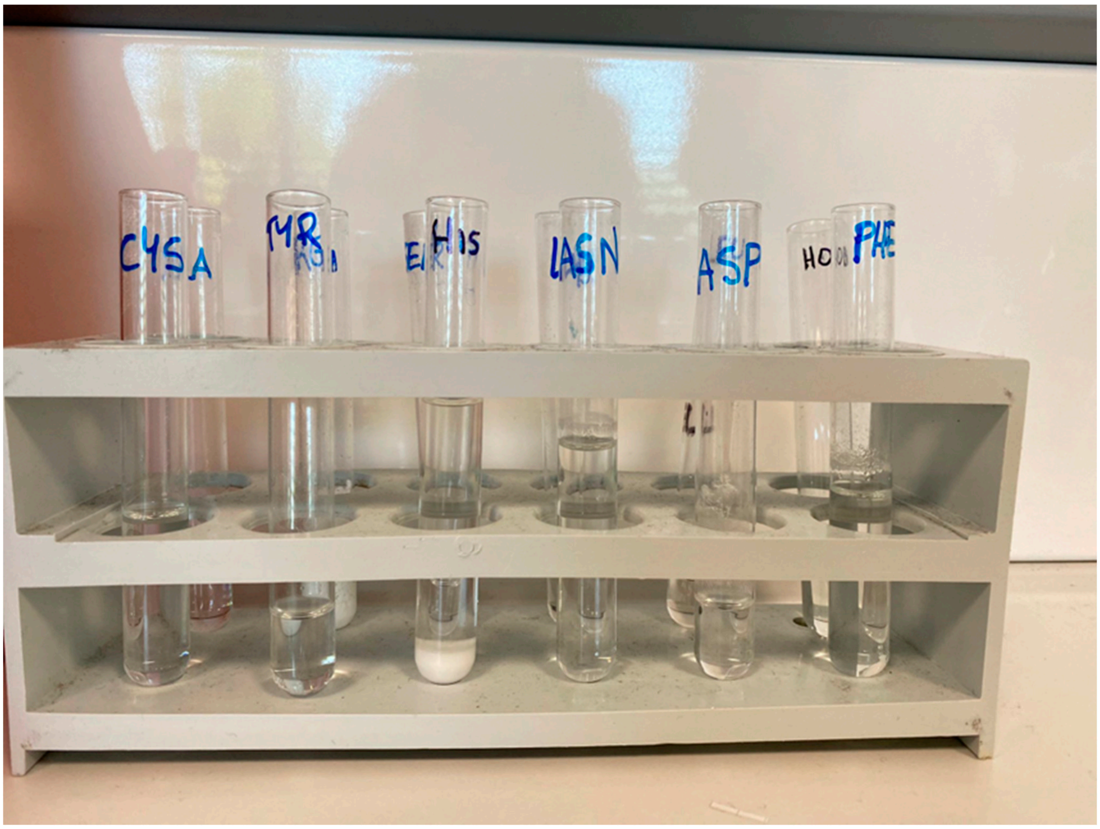

Figure S2. Solubilization efficacy of Fmoc-AA(PG)-OH amino acids in DMM.

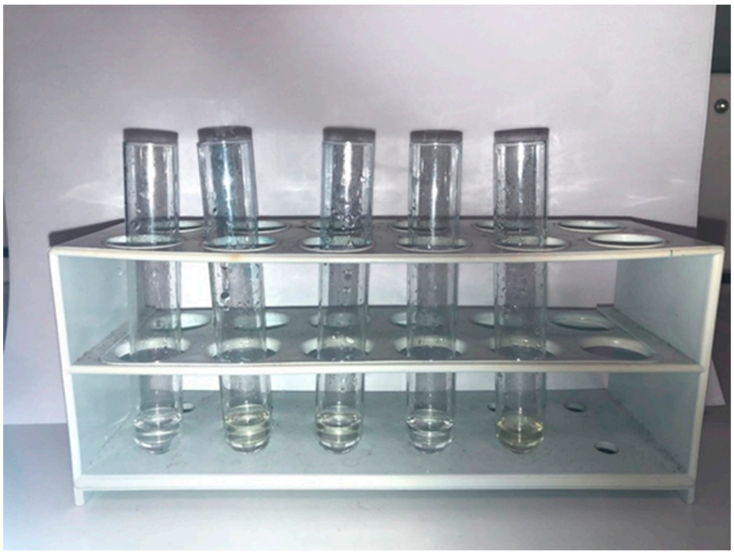

Figure S3. Solubilization efficacy of coupling agents in DMM.

Deprotection kinetics test

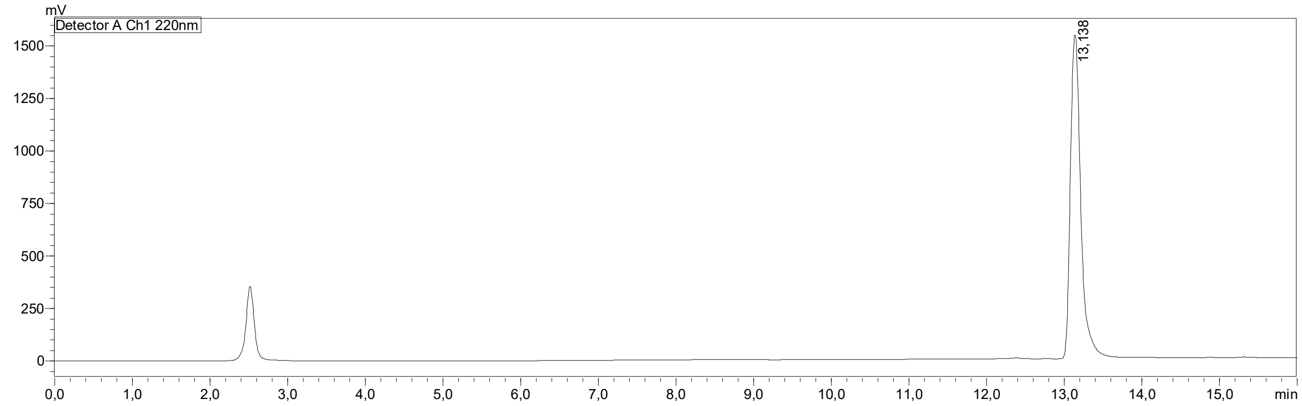

Peak#	Name	Ret. Time	Width	Area	Height	Area%
1	Fmoc-Val-OH	13,138	0,229	14251779	1538817	100,000

Figure S4. Chromatogram of Fmoc-Val-OH deprotection in DMM at t=0 (before base addition).

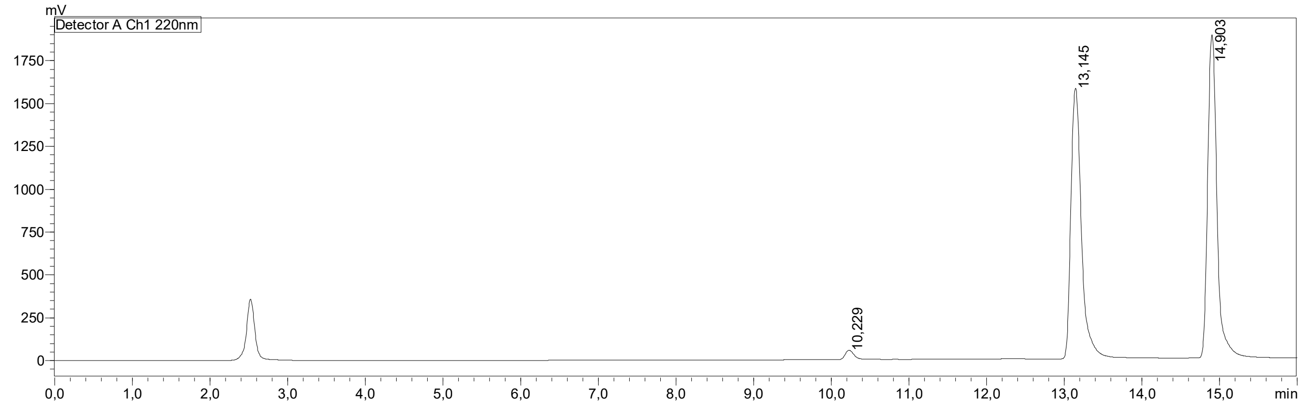

Peak#	Name	Ret. Time	Width	Area	Height	Area%
1	DBF-base adduct	10,229	0,203	418968	55107	1,371
2	Fmoc-Val-OH	13,145	0,230	14715610	1573841	48,143
3	DBF	14,903	0,204	15431657	1883178	50,486

Figure S5. Chromatogram of Fmoc-Val-OH deprotection in DMM at t= 2 min.

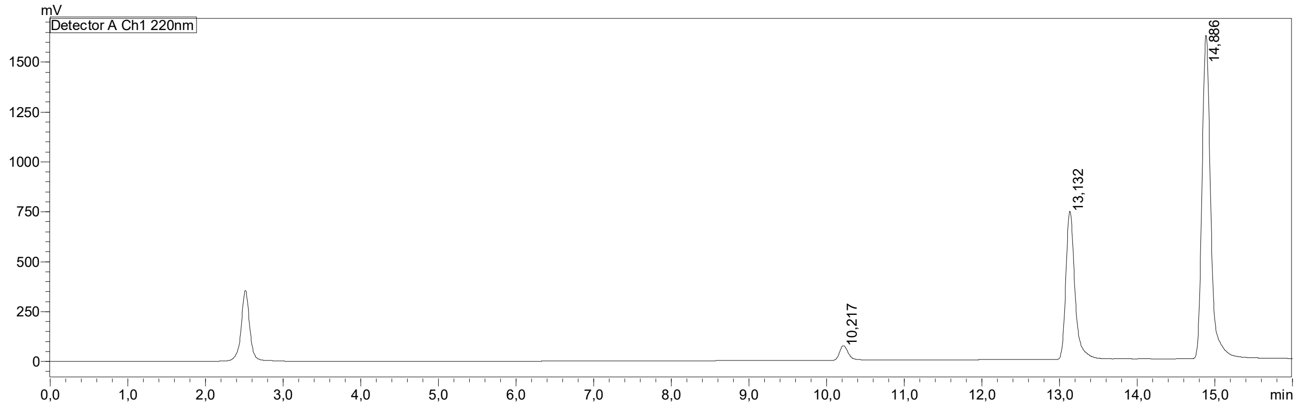

Peak#	Name	Ret. Time	Width	Area	Height	Area%
1	DBF-base adduct	10,217	0,203	548153	72131	2,915
2	Fmoc-Val-OH	13,132	0,205	5982151	742090	31,809
3	DBF	14,886	0,195	12275991	1615485	65,276

Figure S6. Chromatogram of Fmoc-Val-OH deprotection in DMM at t= 4 min.

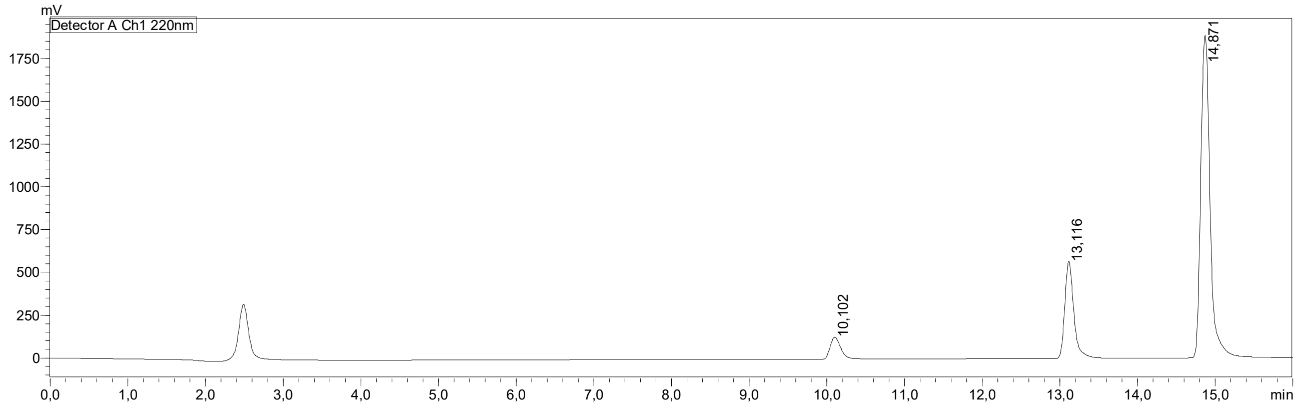

Peak#	Name	Ret. Time	Width	Area	Height	Area%
1	DBF-base adduct	10,102	0,245	1180446	127796	5,613
2	Fmoc-Val-OH	13,116	0,204	4522576	565880	21,505
3	DBF	14,871	0,204	15327048	1880579	72,882

Figure S7. Chromatogram of Fmoc-Val-OH deprotection in DMM at t= 6 min.

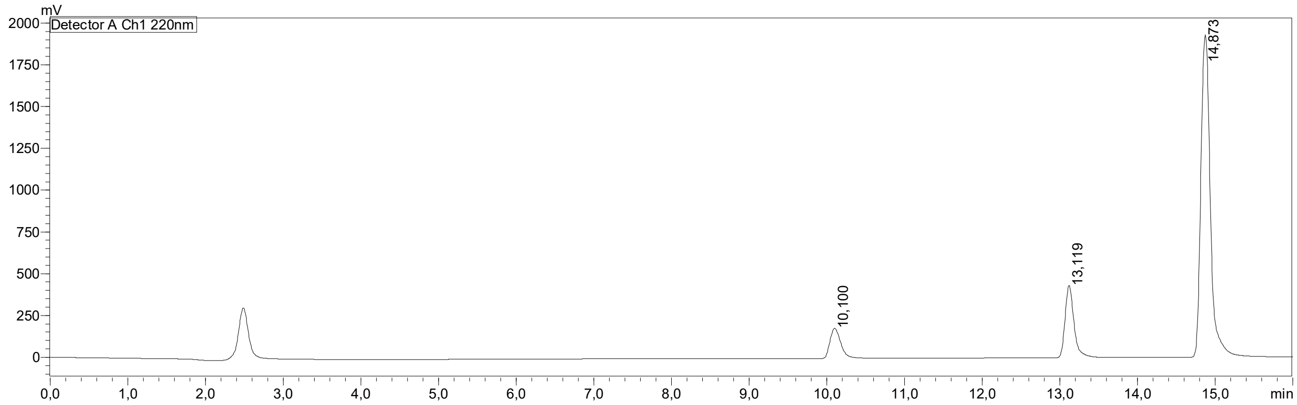

Peak#	Name	Ret. Time	Width	Area	Height	Area%
1	DBF-base adduct	10,100	0,250	1661495	178256	7,973
2	Fmoc-Val-OH	13,119	0,201	3236642	426500	15,531
3	DBF	14,873	0,208	15941492	1923878	76,496

Figure S8. Chromatogram of Fmoc-Val-OH deprotection in DMM at t= 8 min.

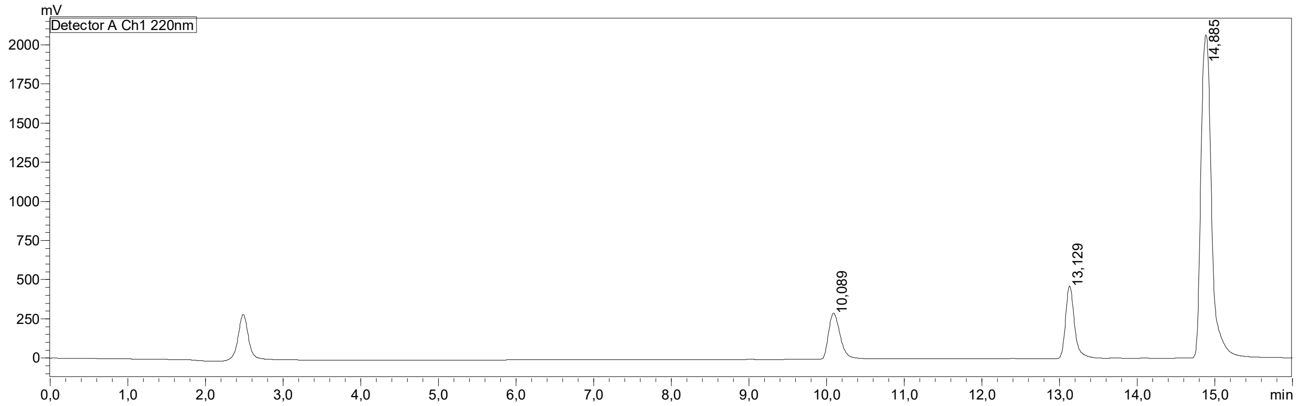

Peak#	Name	Ret. Time	Width	Area	Height	Area%
1	DBF-base adduct	10,089	0,269	2931482	291530	11,458
2	Fmoc-Val-OH	13,129	0,203	3569124	458976	13,950
3	DBF	14,885	0,222	19083894	2057889	74,592

Figure S9. Chromatogram of Fmoc-Val-OH deprotection in DMM at t= 10 min.

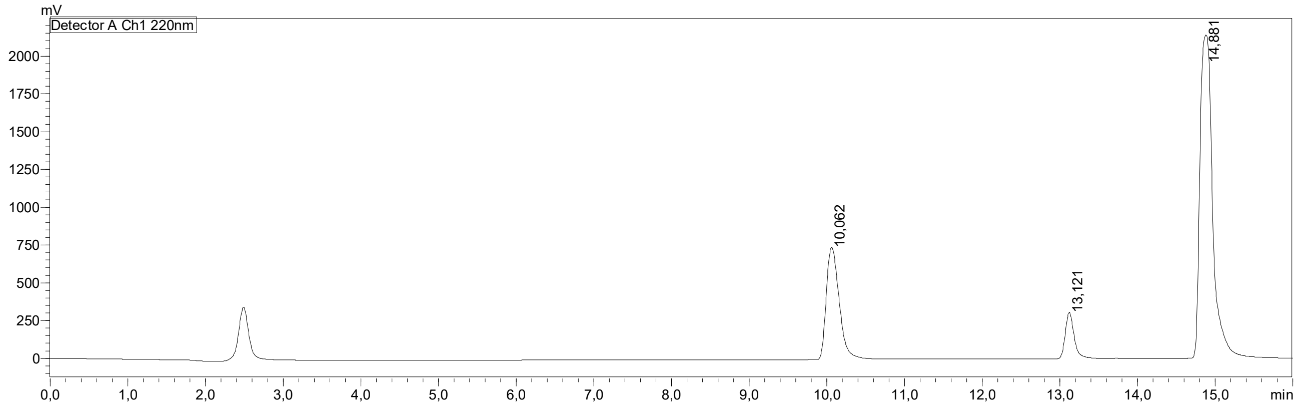

Peak#	Name	Ret. Time	Width	Area	Height	Area%
1	DBF-base adduct	10,062	0,298	8223931	734149	24,040
2	Fmoc-Val-OH	13,121	0,199	2250014	300470	6,577
3	DBF	14,881	0,249	23736102	2132039	69,383

Figure S10. Chromatogram of Fmoc-Val-OH deprotection in DMM at t= 15 min.

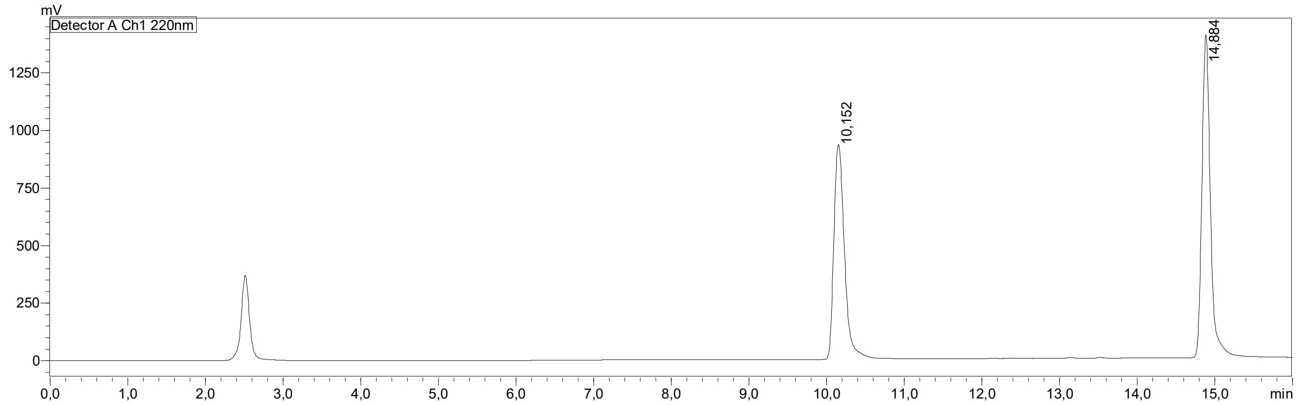

Peak#	Name	Ret. Time	Width	Area	Height	Area%
1	DBF-base adduct	10,152	0,242	8760171	932131	45,734
2	DBF	14,884	0,191	10394613	1398695	54,266

Figure S11. Chromatogram of Fmoc-Val-OH deprotection in DMM at t= 30 min.

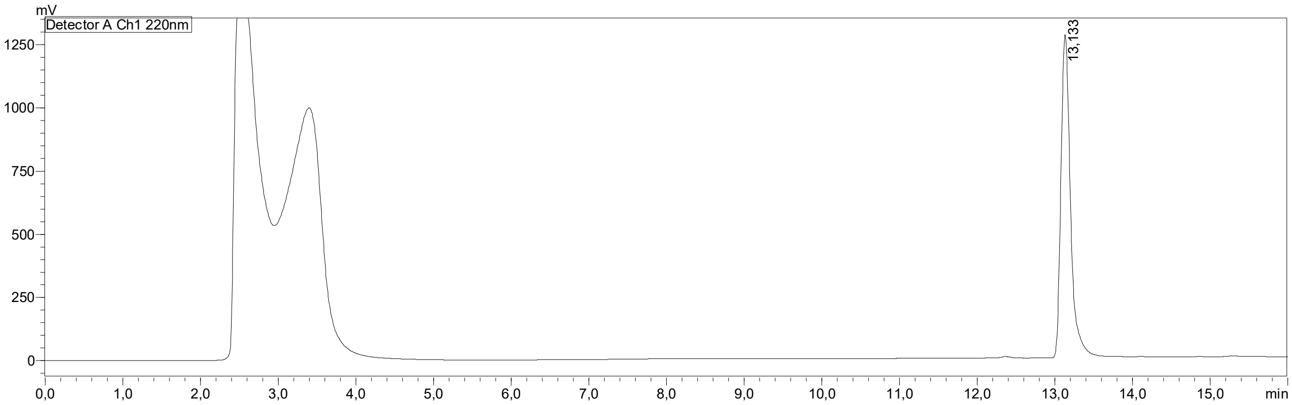

Peak#	Name	Ret. Time	Width	Area	Height	Area%
1	Fmoc-Val-OH	13,133	0,215	10821356	1275772	100,000

Figure S12. Chromatogram of Fmoc-Val-OH deprotection in DMF at t=0 (before base addition).

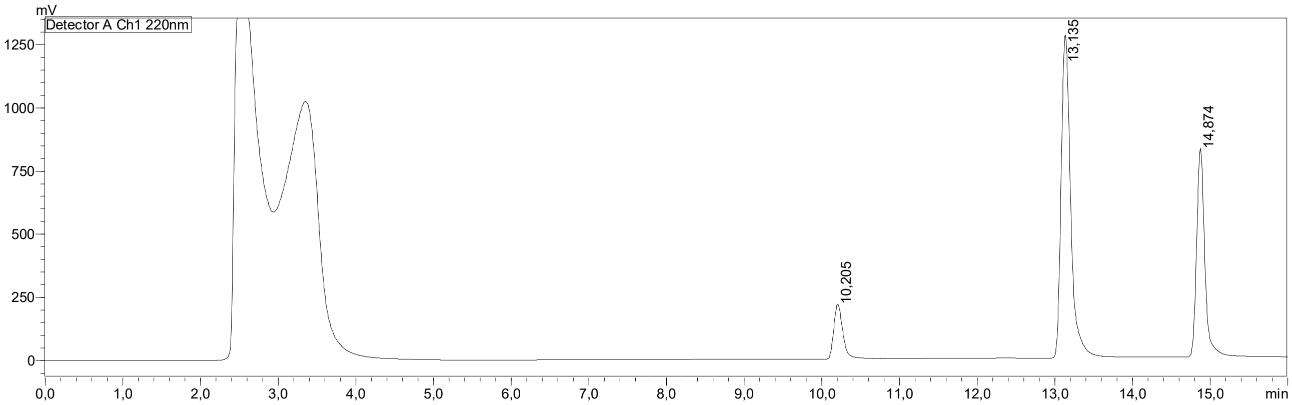

Peak#	Name	Ret. Time	Width	Area	Height	Area%
1	DBF-base adduct	10,205	0,204	1617176	213629	8,921
2	Fmoc-Val-OH	13,135	0,213	10702545	1274616	59,042
3	DBF	14,874	0,185	5807160	822032	32,036

Figure S13. Chromatogram of Fmoc-Val-OH deprotection in DMF at t= 2 min.

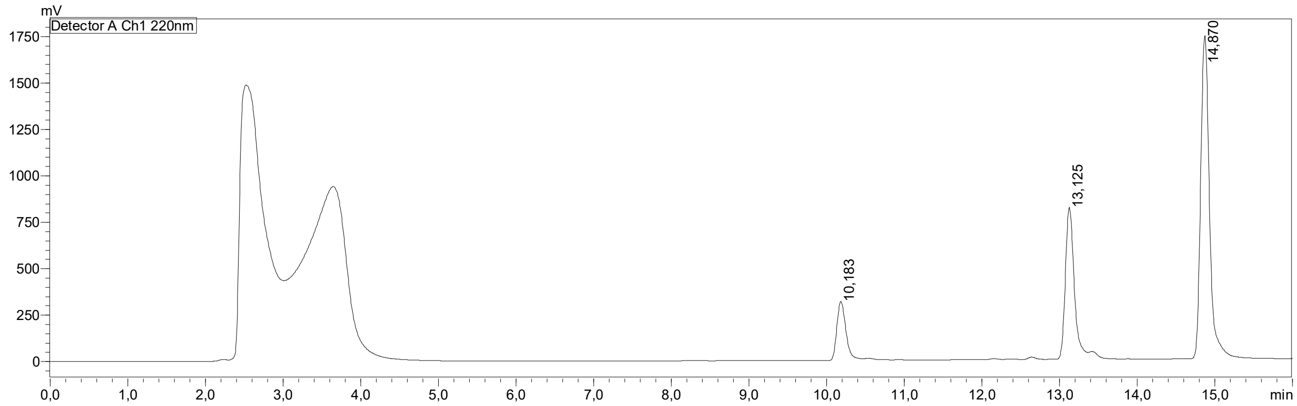

Peak#	Name	Ret. Time	Width	Area	Height	Area%
1	DBF-base adduct	10,183	0,209	2464302	314820	10,872
2	Fmoc-Val-OH	13,125	0,203	6721964	815796	29,657
3	DBF	14,870	0,197	13479233	1737183	59,470

Figure S14. Chromatogram of Fmoc-Val-OH deprotection in DMF at t= 4 min.

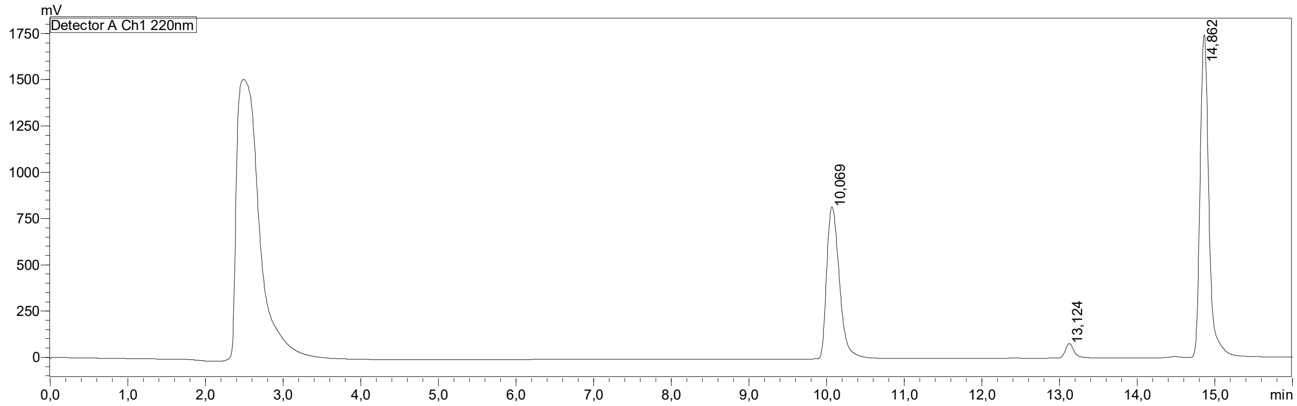

Peak#	Name	Ret. Time	Width	Area	Height	Area%
1	DBF-base adduct	10,069	0,292	9074854	817871	39,383
2	Fmoc-Val-OH	13,124	0,189	511591	74331	2,220
3	DBF	14,862	0,197	13455889	1737182	58,396

Figure S15. Chromatogram of Fmoc-Val-OH deprotection in DMF at t= 6 min.

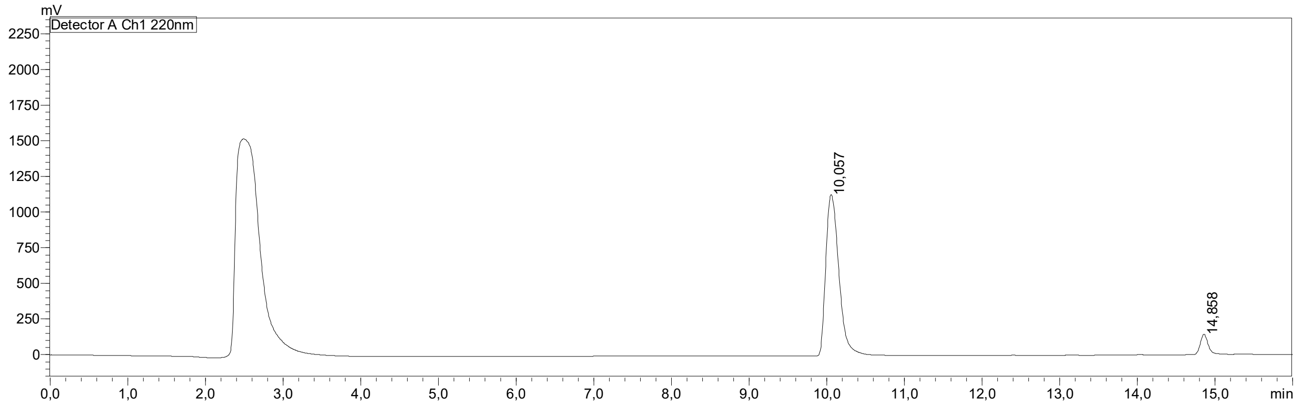

Peak#	Name	Ret. Time	Width	Area	Height	Area%
1	DBF-base adduct	10,057	0,301	13023434	1126822	93,681
2	DBF	14,858	0,176	878511	137028	6,319

Figure S16. Chromatogram of Fmoc-Val-OH deprotection in DMF at t= 8 min.

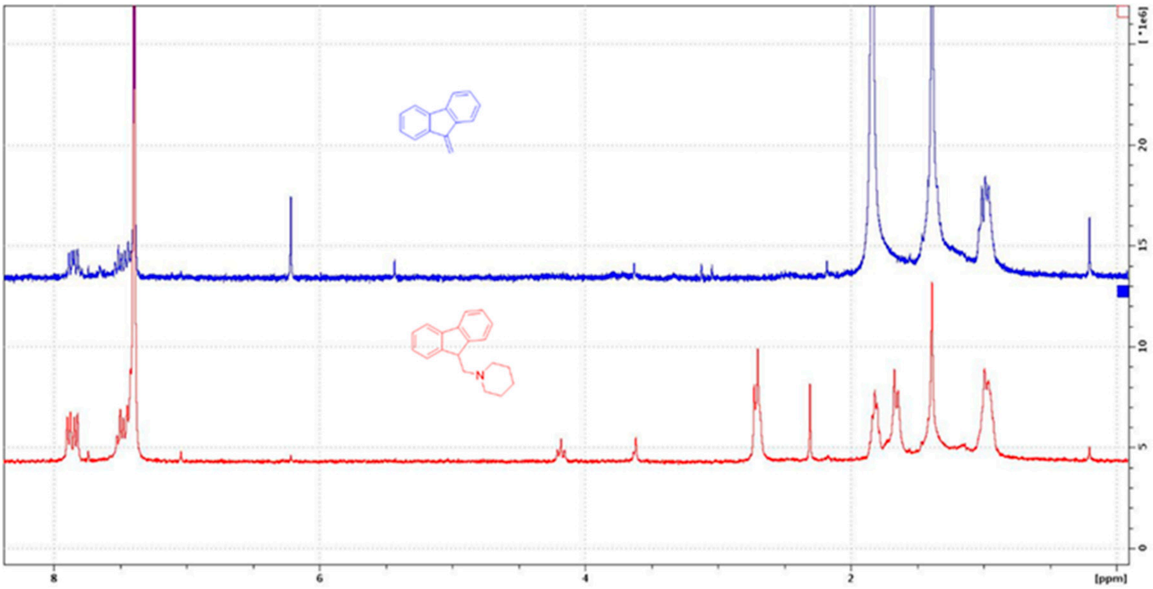

Figure S17. 1H NMR spectrum (400 MHz, DCM-d6) after deprotection with 20% piperidine in DMM of Fmoc-Val-OH; the peaks at 6.45 ppm and aromatic signals between 7.5 and 8.5 ppm correspond to DBF, the peaks at 2.75 ppm and aromatic signals between 7.5 and 8.5 ppm correspond to DBF-base adduct.

Coupling test

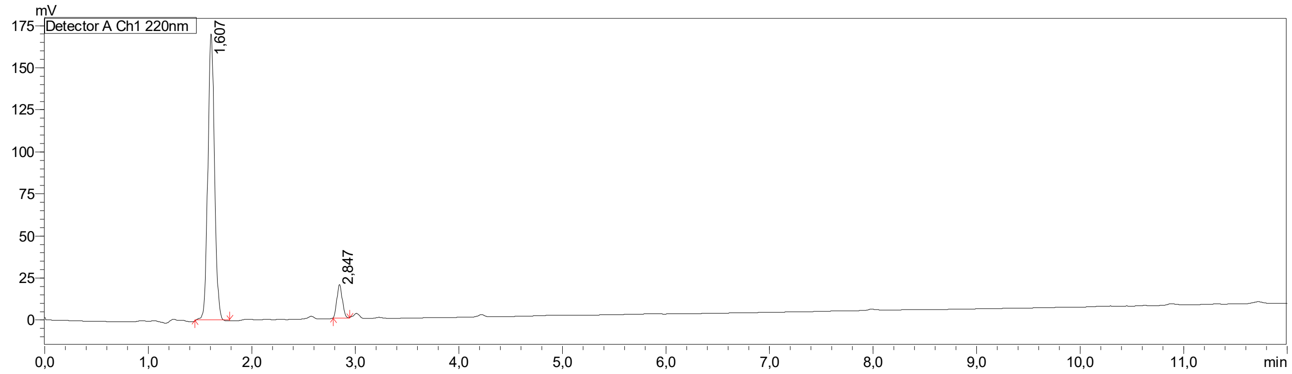

Peak#	Name	Ret. Time	Width	Area	Height	Area%
1	NH_2-Tyr-Ala-OH	1,607	0,123	755632	170105	91,144
2		2,847	0,104	73419	19854	8,856

Figure S18. Chromatogram of NH_2-Tyr-Ala-OH dipeptide synthesized manually in DMM with (HOBt/DIC) (1:1).

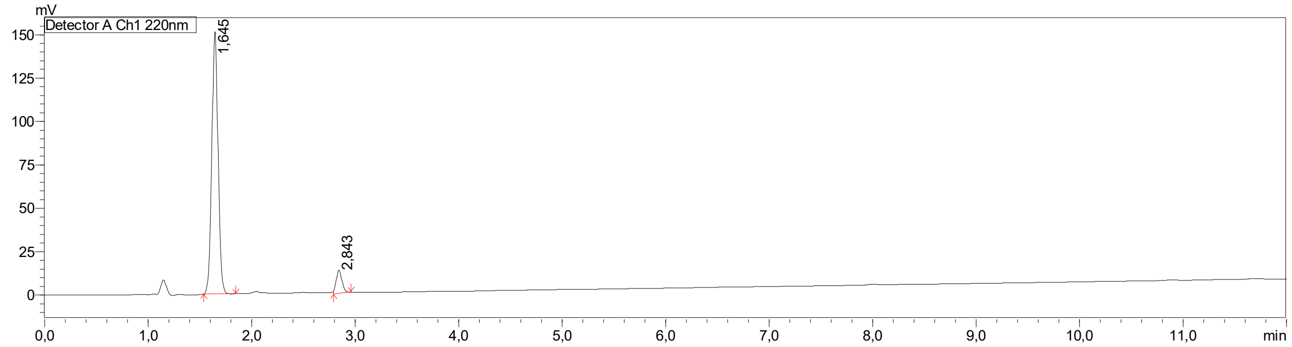

Peak#	Name	Ret. Time	Width	Area	Height	Area%
1	NH_2-Tyr-Ala-OH	1,645	0,117	639199	151103	92,559
2		2,843	0,110	51389	13388	7,441

Figure S19. Chromatogram of NH_2-Tyr-Ala-OH dipeptide synthesized manually in DMM with (HOBt/DIC) (1:1) and purified by HPLC.

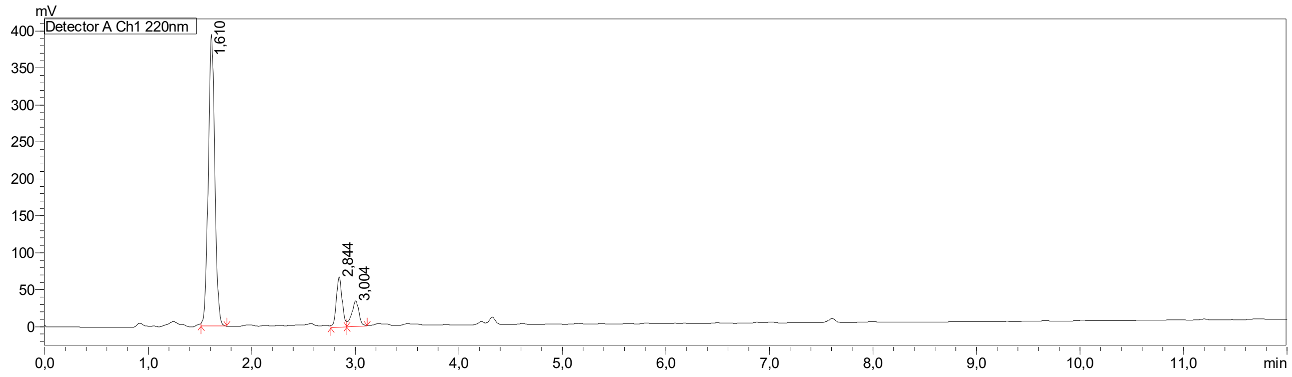

Peak#	Name	Ret. Time	Width	Area	Height	Area%
1	NH_2-Tyr-Ala-OH	1,610	0,122	1725568	394701	79,688
2		2,844	0,110	271185	67999	12,524
3		3,004	0,134	168645	34661	7,788

Figure S20. Chromatogram of NH_2-Tyr-Ala-OH dipeptide synthesized manually in DMF with (HOBt/DIC) (1:1).

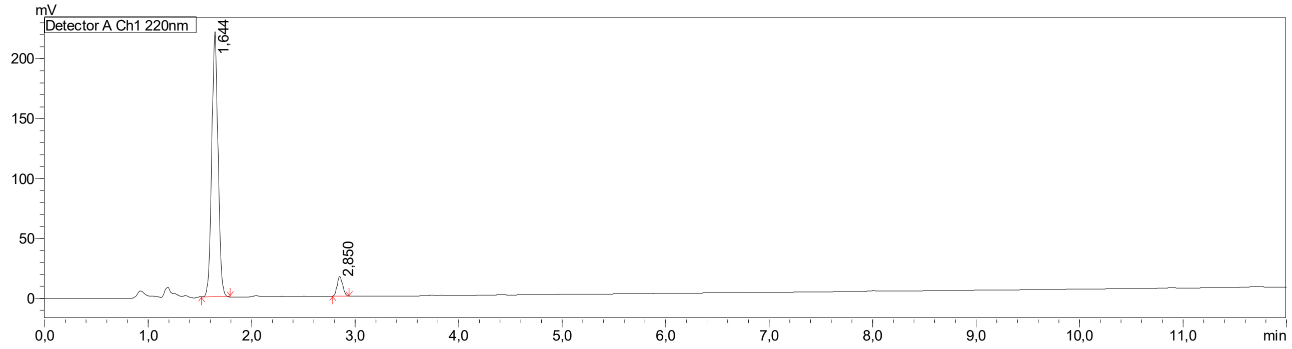

Peak#	Name	Ret. Time	Width	Area	Height	Area%
1	NH_2-Tyr-Ala-OH	1,644	0,118	935849	221057	93,918
2		2,850	0,106	60604	16555	6,082

Figure S21. Chromatogram of NH_2-Tyr-Ala-OH dipeptide synthesized manually in DMF with (HOBt/DIC) (1:1) and purified by HPLC.

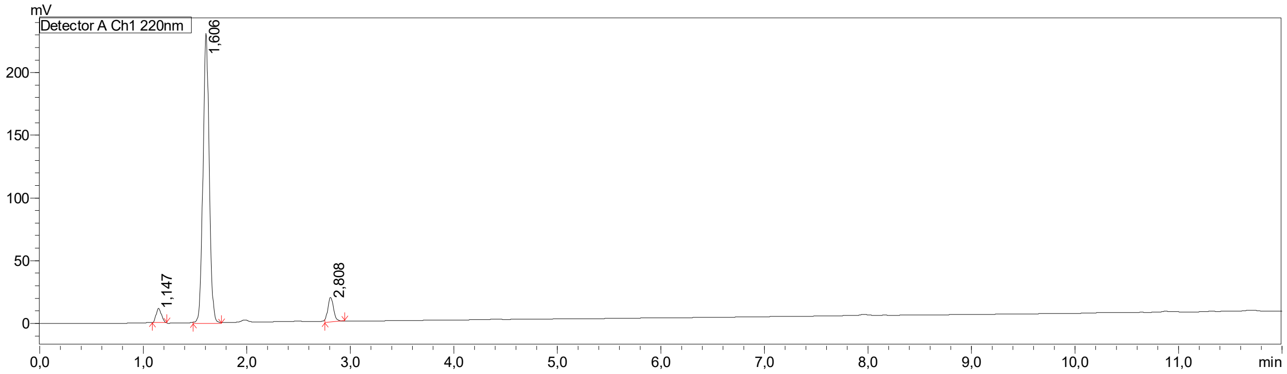

Peak#	Name	Ret.Time	Width	Area	Height	Area%
1		1,147	0,105	40362	11354	3,700
2	NH_2-Tyr-Ala-OH	1,606	0,117	974444	231592	89,326
3		2,808	0,111	76085	19778	6,975

Figure S22. Chromatogram of NH_2-Tyr-Ala-OH dipeptide synthesized manually in DMM with (OxymaPure/DIC) (1:2).

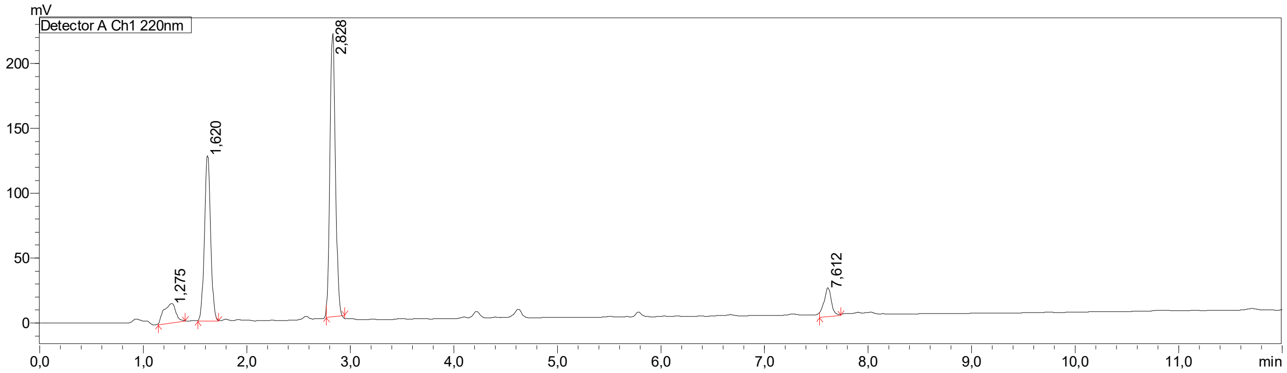

Peak#	Name	Ret. Time	Width	Area	Height	Area%
1		1,275	0,204	124707	15031	7,906
2	NH_2-Tyr-Ala-OH	1,620	0,115	530833	127712	33,651
3		2,828	0,106	808110	218683	51,229
4		7,612	0,140	113805	22243	7,214

Figure S23. Chromatogram of NH_2-Tyr-Ala-OH dipeptide synthesized manually in DMF with (OxymaPure/DIC) (1:2).

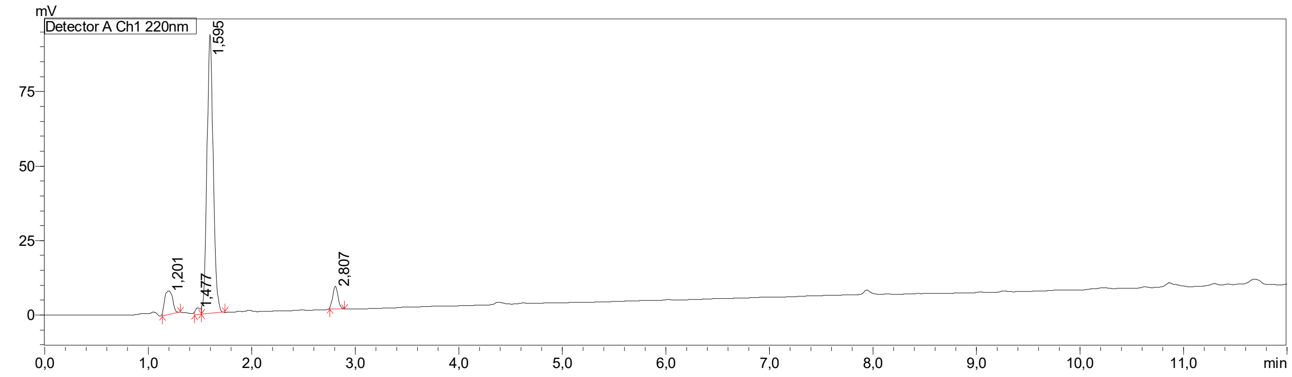

Peak#	Name	Ret. Time	Width	Area	Height	Area%
1		1,201	0,137	42110	7805	8,921
2		1,477	0,345	8164	2244	1,729
3	NH_2-Tyr-Ala-OH	1,595	0,117	394523	93706	83,577
4		2,807	0,102	27251	7579	5,773

Figure S24. Chromatogram of NH_2-Tyr-Ala-OH dipeptide synthesized manually in DMF with (OxymaPure/DIC) (1:2) and purified by HPLC.

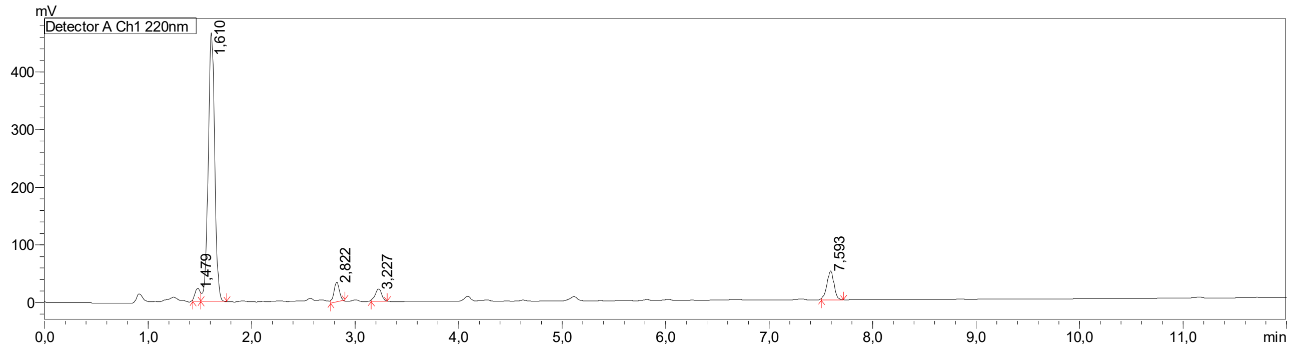

Peak#	Name	Ret. Time	Width	Area	Height	Area%
1		1,479	0,133	79753	22441	3,112
2	NH_2-Tyr-Ala-OH	1,610	0,120	2022168	465661	78,901
3		2,822	0,107	120938	33335	4,719
4		3,227	0,129	98232	21902	3,833
		7,593	0,132	241836	50258	9,436

Figure S25. Chromatogram of NH_2-Tyr-Ala-OH dipeptide synthesized microwave in DMM with (OxymaPure/DIC) (1:2) (3 eq. 2 x 10 min).

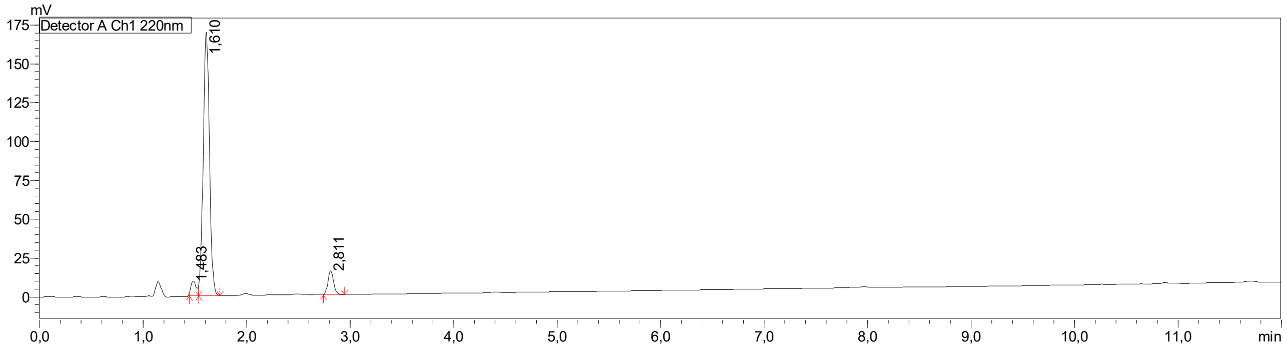

Peak#	Name	Ret. Time	Width	Area	Height	Area%
1		1,483	0,118	36912	9599	4,558
2	NH_2-Tyr-Ala-OH	1,610	0,117	710423	169838	87,716
3		2,811	0,109	62577	15594	7,726

Figure S26. Chromatogram of NH_2-Tyr-Ala-OH dipeptide synthesized microwave in DMM with (OxymaPure/DIC) (1:2) (3 eq. 2 x 10 min) and purified by HPLC.

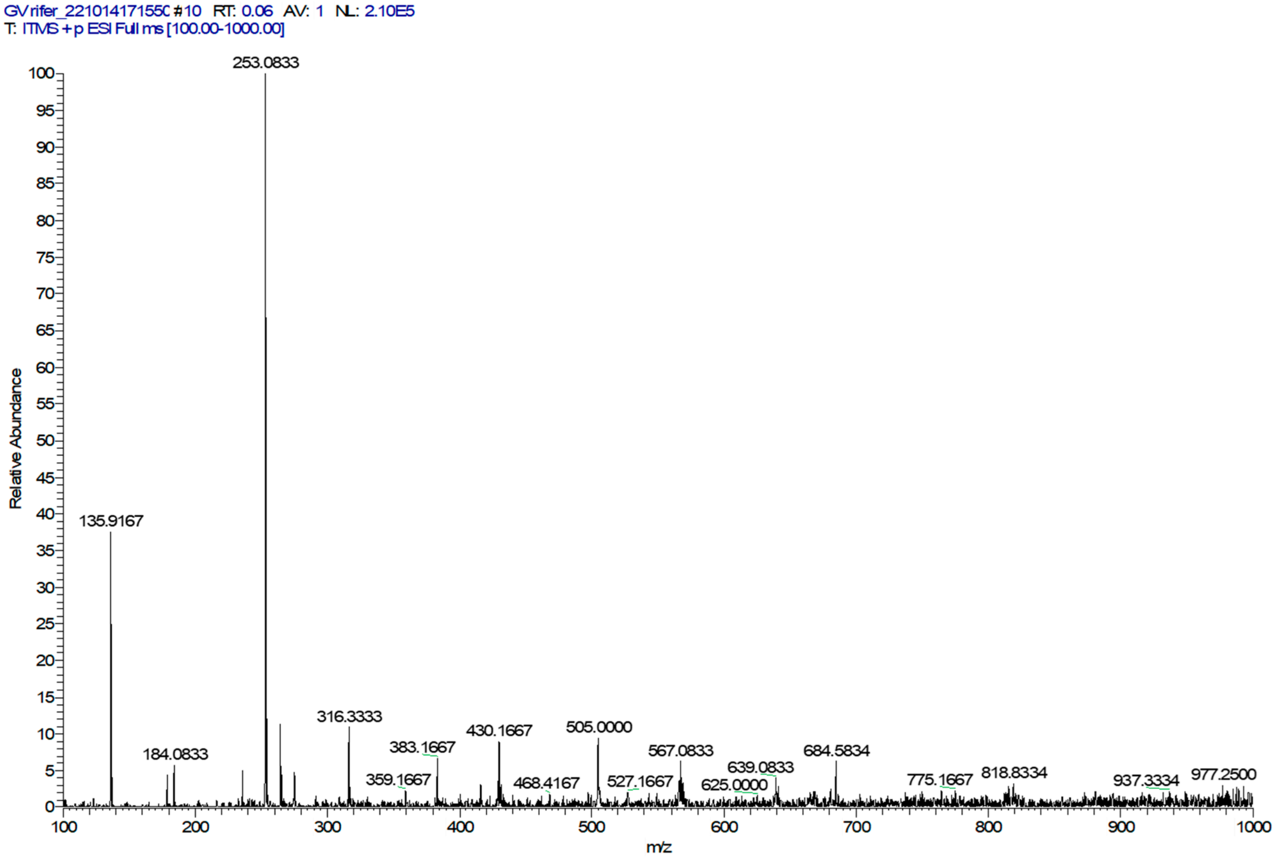

Figure S27. ESI-MS of NH_2-Tyr-Ala-OH dipeptide ion [M+H]+ (253.0833 Da)

Racemization study

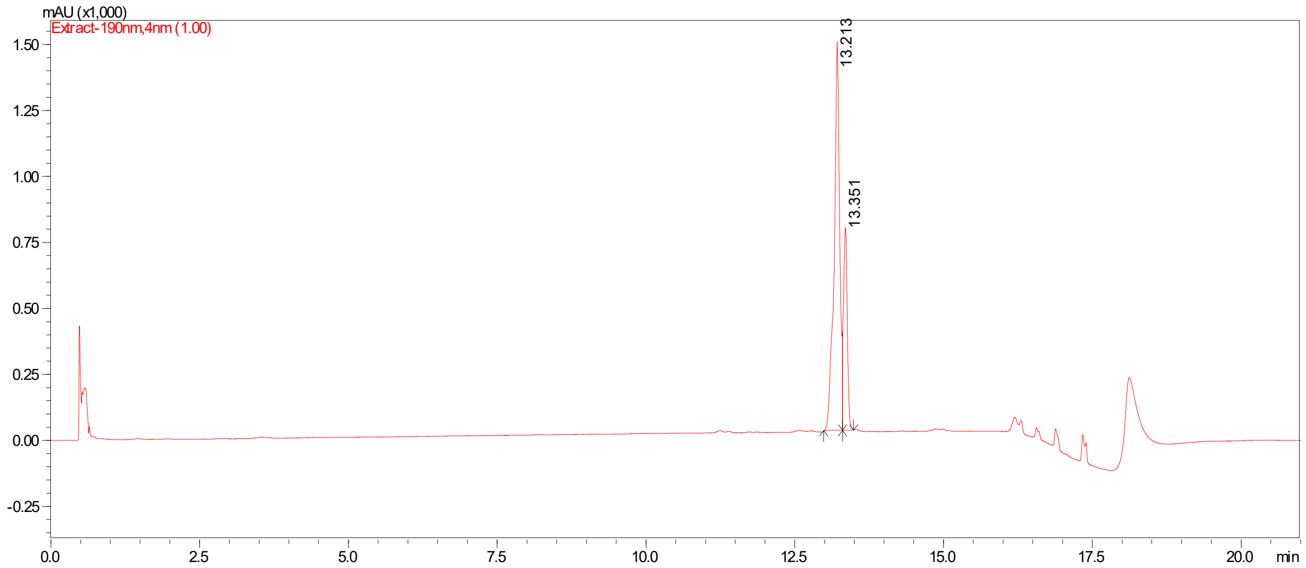

Peak#	Name	Ret. Time	Width	Area	Height	Area%
1	Fmoc-Phg-Pro-NH_2	13.213	0.086	9539379	1463542	73.9107
2	Fmoc-D-Phg-Pro-NH_2	13.351	0.081	3367245	760246	26.0893

Figure S28. Chromatogram of Fmoc-Phg-Pro-NH_2 dipeptide synthesized microwave in DMM

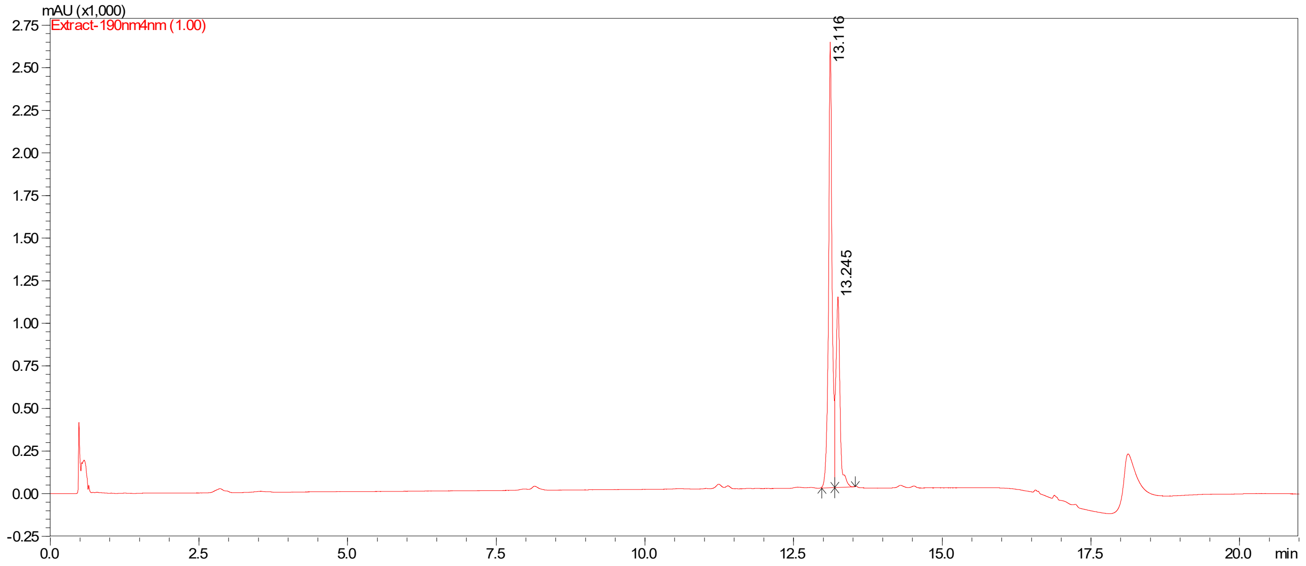

Peak#	Name	Ret. Time	Width	Area	Height	Area%
1	Fmoc-Phg-Pro-NH_2	13.116	0.054	10923289	2597350	67.2107
2	Fmoc-D-Phg-Pro-NH_2	13.245	0.079	5329020	1114877	32.7893

Figure S29. Chromatogram of Fmoc-Phg-Pro-NH_2 dipeptide synthesized manually in DMF

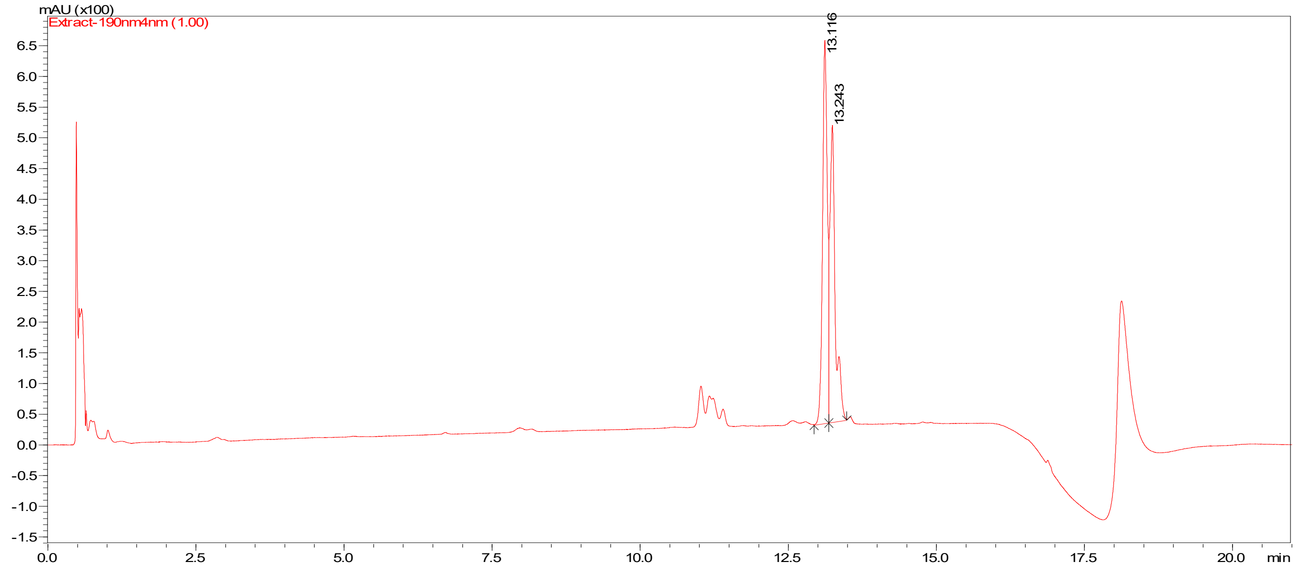

Peak#	Name	Ret. Time	Width	Area	Height	Area%
1	Fmoc-Phg-Pro-NH_2	13.116	0.095	3437442	620302	52.9032
2	Fmoc-D-Phg-Pro-NH_2	13.243	0.000	3060166	481172	47.0968

Figure S30. Chromatogram of Fmoc-Phg-Pro-NH_2 dipeptide synthesized microwave in DMF

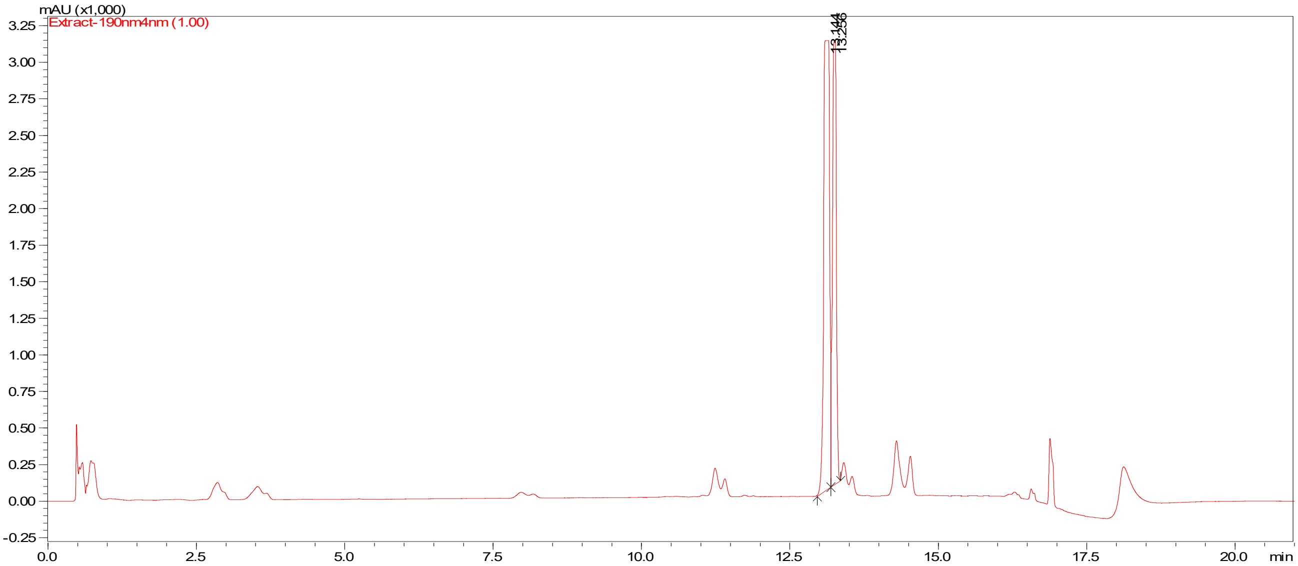

Peak#	Name	Ret. Time	Width	Area	Height	Area%
1	Fmoc-Phg-Pro-NH_2	13.144	0.105	21168928	3066497	60.3108
2	Fmoc-D-Phg-Pro-NH_2	13.256	0.069	13930797	3034445	39.6892

Figure S31. Chromatogram of Fmoc-Phg-Pro-NH_2 dipeptide synthesized manually in DMM

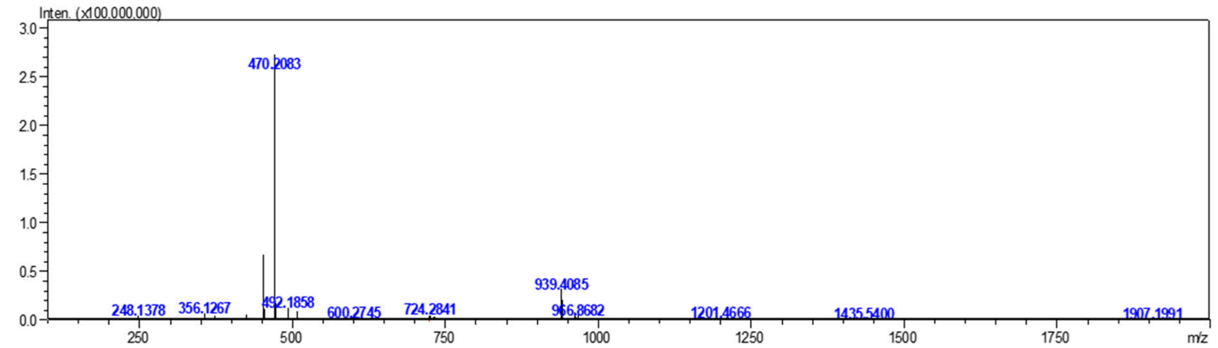

Figure S32. ESI-MS of Fmoc-Phg-Pro-NH2 dipeptide ion [M+H]+ (470.2083 Da)

Green SPPS

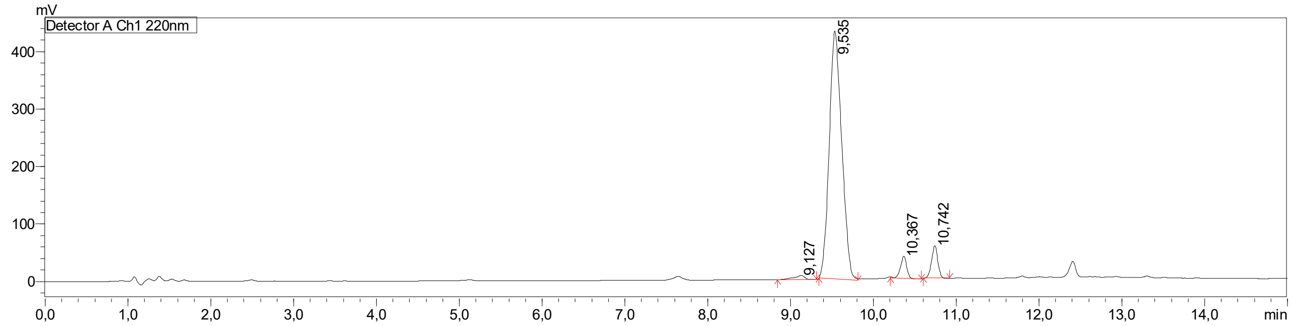

Peak#	Name	Ret. Time	Width	Area	Height	Area%
1	Des-Aib	9,127	0,213	70358	7140	1,389
2	Aib-enkephalin	9,535	0,293	4519352	431118	89,199
3	Wang linker adduct + tBu	10,367	0,140	198111	38556	3,910
4	Des-Aib + tBu + TFA	10,742	0,142	278759	55501	5,502

Figure S33. Chromatogram of Aib- enkephalin synthesized on Wang PS resin in DMF

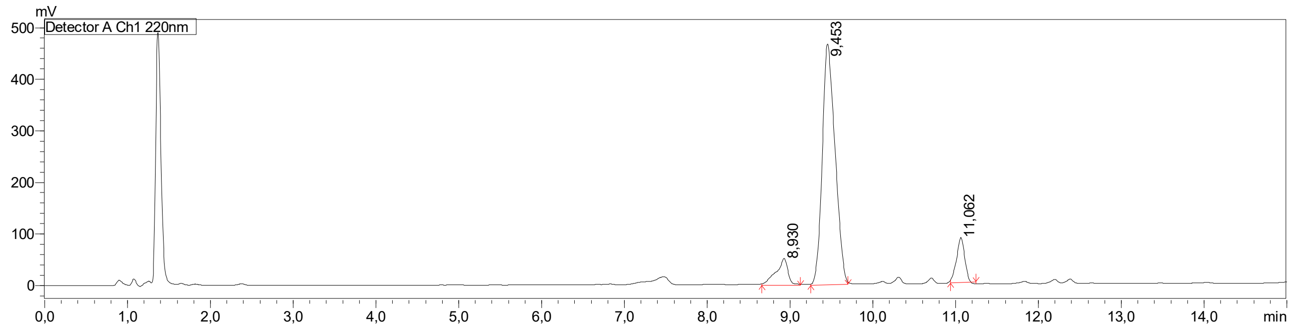

Peak#	Name	Ret. Time	Width	Area	Height	Area%
1	Des-Aib	8,930	0,215	530264	52072	8,655
2	Aib-enkephalin	9,453	0,302	5008932	466523	81,759
3	Dimer Aib-enkephalin + formyl	11,062	0,189	587247	87345	9,585

Figure S34. Chromatogram of Aib- enkephalin synthesized on Wang PS resin in DMM

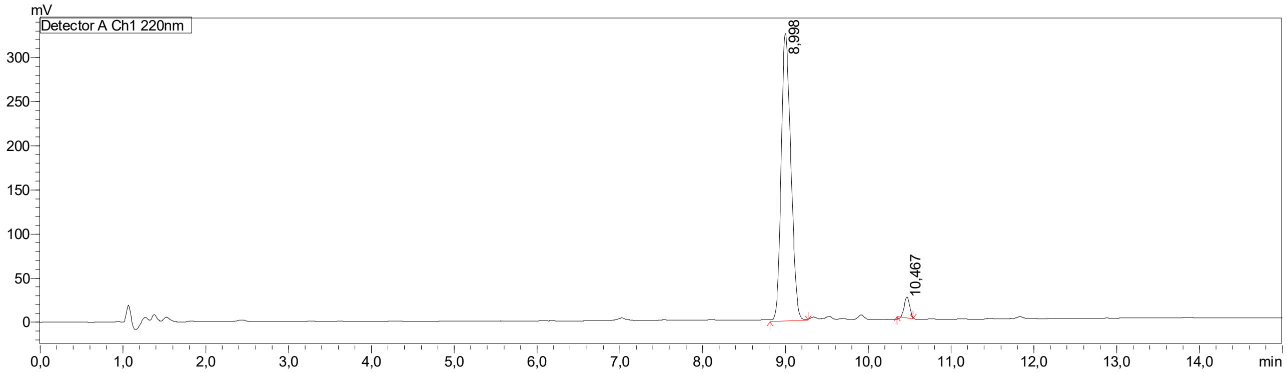

Peak#	Name	Ret. Time	Width	Area	Height	Area%
1	Aib-enkephalin	8,998	0,225	2656080	325946	94,877
2	Des-Aib + tBu + TFA	10,467	0,141	143431	25747	5,123

Figure S35. Chromatogram of Aib- enkephalin synthesized on Rink-Amide CM resin in DMF

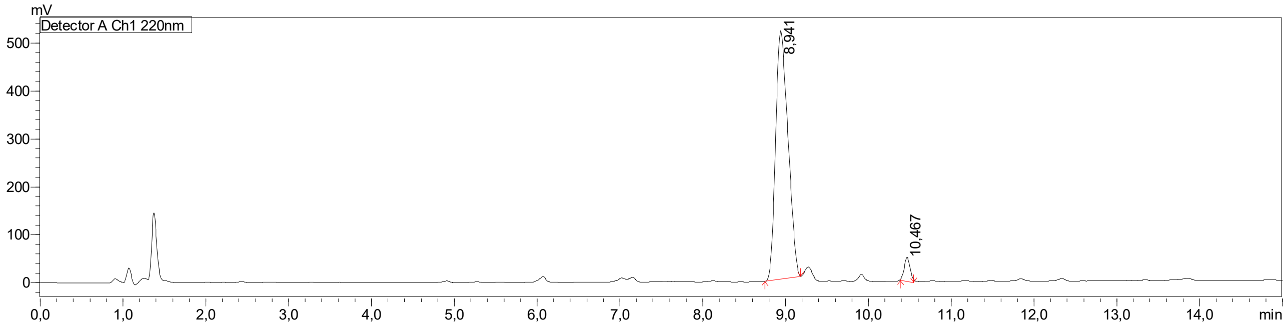

Peak#	Name	Ret. Time	Width	Area	Height	Area%
1	Aib-enkephalin	8,941	0,299	5392569	517778	95,597
3	Des-Aib+tBu+TFA	10,467	0,140	248392	50199	4,403

Figure S36. Chromatogram of Aib- enkephalin synthesized on Rink-Amide CM resin in DMM

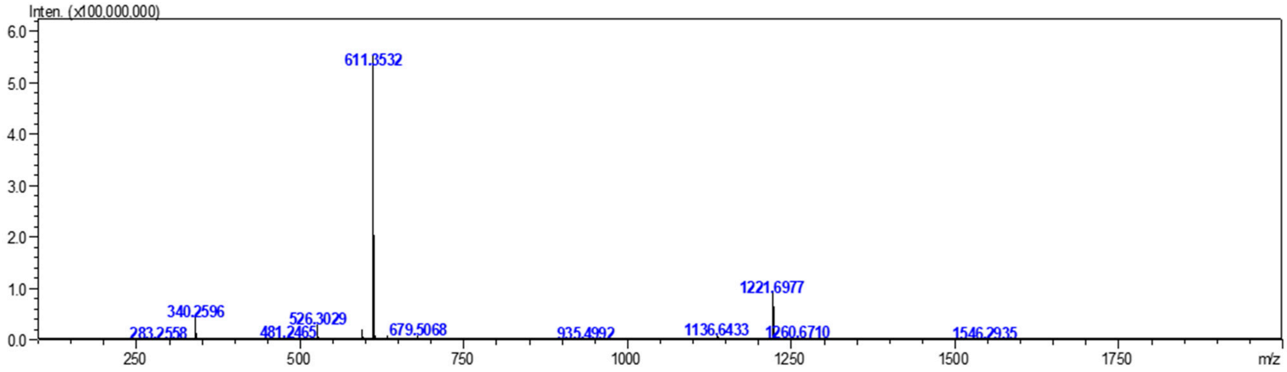

Figure S37. ESI-MS of Aib- enkephalin ion [M+H]+ (611.3532 Da)

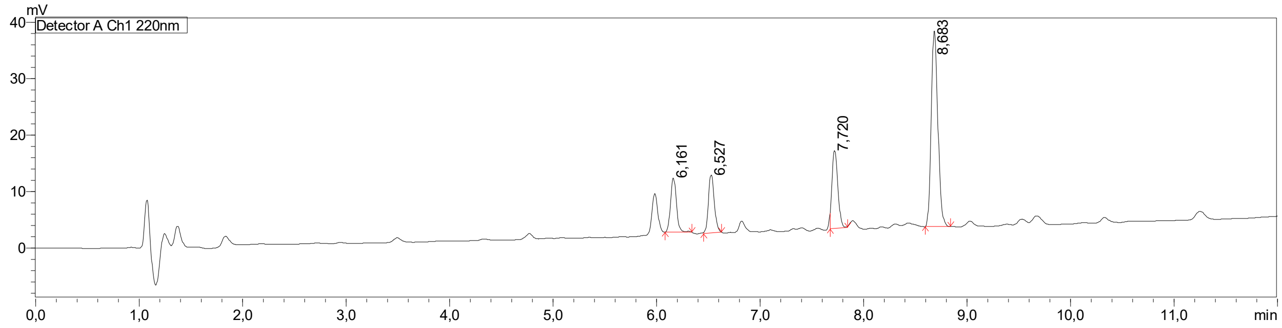

Peak#	Name	Ret. Time	Width	Area	Height	Area%
1	Des-Aib-Gln	6,161	0,110	37987	9633	13,231
2	Des-Asp	6,527	0,110	40576	10226	14,132
3	Des-Val	7,720	0,114	54264	13765	18,900
4	Aib-ACP	8,683	0,124	154288	34533	53,737

Figure S38. Chromatogram of Aib-ACP synthesized in DMF

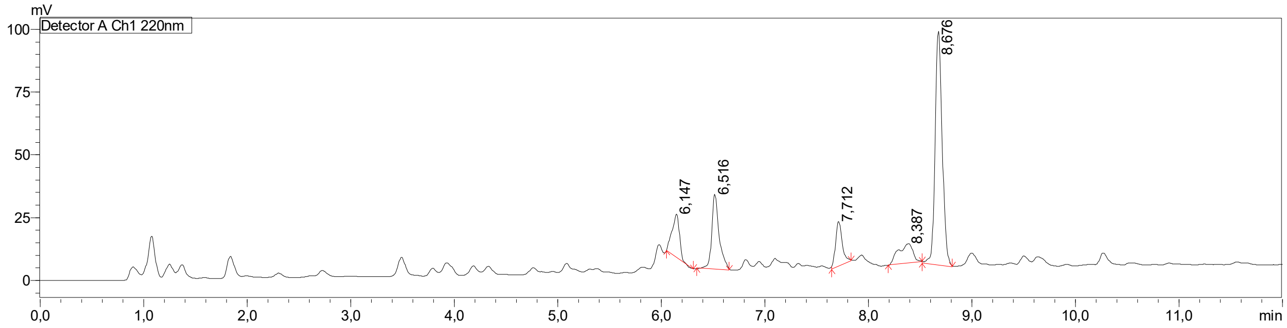

Peak#	Name	Ret. Time	Width	Area	Height	Area%
1	Des-Aib-Gln	6,147	0,140	81445	17126	9,792
2	Des-Asp	6,516	0,127	147263	29768	17,706
3	Des-Val	7,712	0,120	79572	17741	9,567
4	Des-Aib	8,387	0,255	76855	7767	9,240
5	Aib-ACP	8,676	0,131	446593	93089	53,695

Figure S39. Chromatogram of Aib-ACP synthesized in DMM

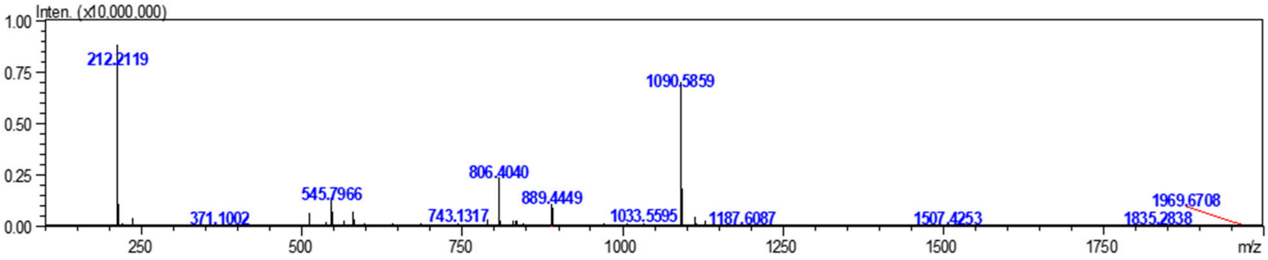

Figure S40. ESI-MS of Aib-ACP ion [M+H]+ (1090.5859 Da)

Recycling of decapeptide SPPS waste stream

The scale of the decapeptide was 0.015 mmol. The amount of crude product obtained from the synthesis is 0.0996g (for all cases)

$PMI = mass\ of\ materials$ (g) $/mass\ of\ isolated\ product$ (g)

Specifically, mass of materials includes:

- Starting materials: Fmoc-AA-OH, DIC, Oxyma Pure®, resin, cleavage cocktail
- Solvents (DMF or DMM and Et_2O for crude peptide precipitation)
- Base (piperidine)

Table S4. Overview of starting materials used for decapeptide (Aib-ACP) and their total mass.

	MW (g/mol)	d (g/mL)	eq	Volume (mL)	Concentration (mol/L)	Mass (g)
Fmoc-Aib-OH	325.4		3		0.1	0.187
Fmoc-Gly-OH	297.3		3		0.1	0.087
Fmoc-Asn(Trt)-OH	596.7		3		0.1	0.174
Fmoc-Tyr(tBu)-OH	459.6		3		0.1	0.134
Fmoc-Asp(tBu)-OH	411.5		3		0.1	0.120
Fmoc-Ile-OH	353.4		3		0.1	0.203
Fmoc-Val-OH	339.4		3		0.1	0.099
Fmoc-Gln(Trt)-OH	610.7		3		0.1	0.178
OxymaPure	142.1		3		0.5	0.408
DIC	126.2	0.815	6		0.5	0.080
Piperidine	85.1	0.862		14.548	2.0	12.540

Cleavage and precipitation

TFA	114.2	1.489		4.75		7.070
TIPS	158.36	0.773		0.125		0.0966
H_2O	18.02	0.997		0.125		0.124
Et_2O	102.17	0.725		75.0		54.370
					**Total:**	**75.87**

Table S5. Overview of solvents used for decapeptide (Aib-ACP) and their total mass.

DMF

	MW (g/mol)	d (g/mL)	Volume (mL)	Mass (g)
DMF couplings	73.09	0.944	44.28	41.80
DMF washings after couplings	73.09	0.944	90.00	84.96
DMF deprotection	73.09	0.944	40.45	38.18
DMF washings after deprotection	73.09	0.944	156.00	147.26
			**Total:**	**312.20**

DMM

	MW (g/mol)	d (g/mL)	Volume (mL)	Mass (g)
DMM couplings	162.23	0.90	44.28	39.85
DMM washings after couplings	162.23	0.90	90.00	81
DMM deprotection	162.23	0.90	40.45	36.40
DMM washings after deprotection	162.23	0.90	156.00	140.4
			**Total:**	**297.65**

Table S6. Total mass of materials used for SPPS without recycling and PMI

	Solvent	
	DMF	DMM
Total mass of starting materials (g)	75.87	75.87
Total mass of solvents (g)	312.20	297.65
**Total (g)**	**388.07**	**373.52**

PMI

$$PMI = \frac{\sum mass\ of\ materials\ (g)}{mass\ of\ isolated\ product\ (g)}$$

$$PMI\ of\ synthesis\ in\ DMF = \frac{388.07}{0.0996} = 3896.28$$

$$PMI\ of\ synthesis\ in\ DMM = \frac{373.52}{0.0996} = 3750.20$$

Table S7. Total mass of recovered solvent (DMM) and PMI with recycling of synthesis in DMM

	Solvent
	DMM
Total mass of solvent (g)	297.65
Total mass of recycled solvent (g)	237

$$PMI\ (with\ recycling) = \frac{\sum mass\ of\ materials - \sum mass\ of\ recovered\ materials}{mass\ of\ isolated\ product}$$

$$PMI\ (with\ recycling) = \frac{373.52 - 237}{0.0996} = 1370.68$$

Figure S41. (a) ^1H NMR spectrum (CD2Cl2, 400 MHz, 298 K) of DMM recovered from

distillation processes; (b) ^{1}H NMR spectrum of DMM

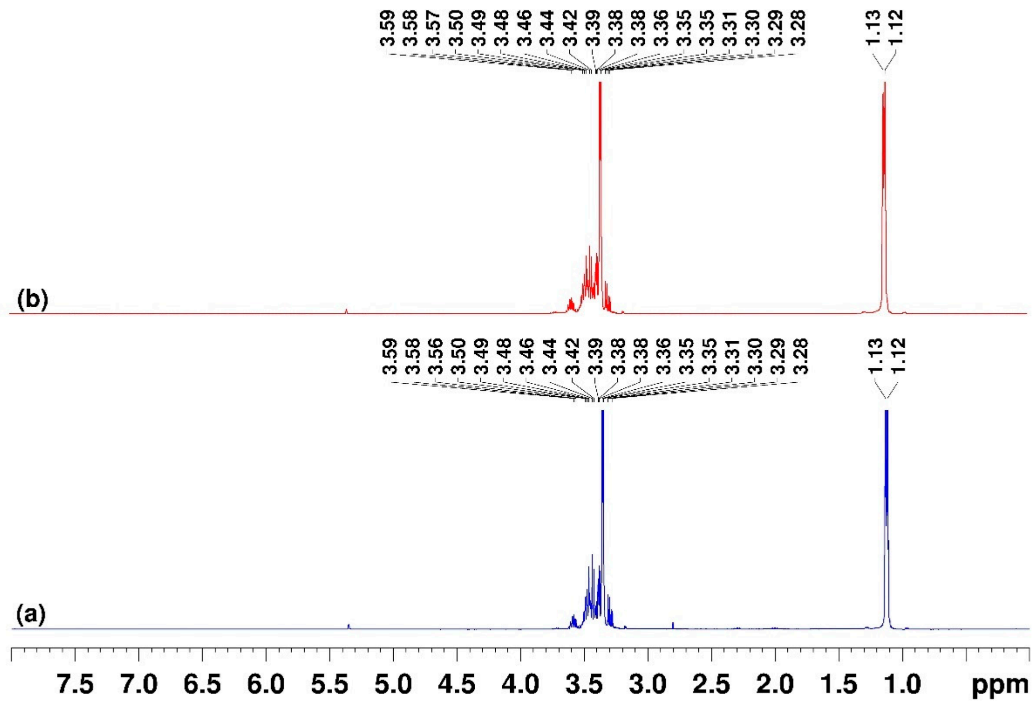

Supplement: Supplementary file 1 [file pharmaceutics-15-01773-s001.zip › pharmaceutics-2451357-supplementary.pdf]
